# Synthesis of new binary trimethoxyphenylfuran pyrimidinones as proficient and sustainable corrosion inhibitors for carbon steel in acidic medium: experimental, surface morphology analysis, and theoretical studies

**DOI:** 10.1186/s13065-024-01280-6

**Published:** 2024-09-20

**Authors:** Hajar A. Ali, Ahmed. A. El-Hossiany, Ashraf S. Abousalem, Mohamed A. Ismail, Abd El-Aziz S. Fouda, Eslam A. Ghaith

**Affiliations:** 1 Chemistry Department, Faculty of Science, Mansoura University, Mansoura, 35516 Egypt; 2 Delta for Fertilizers and Chemical Industries, Talkha, Egypt; 3 Operations Department, Quality Control Laboratory, Jotun, Egypt

**Keywords:** Corrosion inhibition, Carbon steel, Pyrimidinone derivatives, Quantum chemical calculations

## Abstract

In this study, synthesis and assessment of the corrosion inhibition of four new binary heterocyclic pyrimidinones on CS in 1.0 M hydrochloric acid solutions at various temperatures (30–50 °C) were investigated. The synthesized molecules were designed and synthesized through Suzuki coupling reaction, the products were identified as 5-((5-(3,4,5-trimethoxyphenyl)furan-2-yl)methylene)pyrimidine-2,4,6(1*H*,3*H*,5*H*)-trione (**HM-1221**), 2-thioxo-5-((5-(3,4,5-trimethoxyphenyl)furan-2-yl)methylene)dihydropyrimidine-4,6(1*H*,5*H*)-dione (**HM-1222**), 1,3-diethyl-2-thioxo-5-((5-(3,4,5-trimethoxyphenyl)furan-2-yl)methylene)dihydropyrimidine-4,6(1*H*,5*H*)-dione (**HM-1223**) and 1,3-dimethyl-5-((5-(3,4,5-trimethoxyphenyl)furan-2-yl)methylene)pyrimidine-2,4,6(1*H*,3*H*,5*H*)-trione (**HM-1224**). The experiments include weight loss measurements (WL), electrochemical impedance spectroscopy (EIS) and potentiodynamic polarization (PDP). From the measurements, it can be shown that the inhibition efficiency (*η*) of these organic derivatives increases with increasing the doses of inhibitors. The highest *η* recorded from EIS technique were 89.3%, 90.0%, 92.9% and 89.7% at a concentration of 11 × 10^−6^ M and 298 K for **HM-1221**, **HM-1222**, **HM-1223**, and **HM-1224**, respectively. The adsorption of the considered derivatives fit to the Langmuir adsorption isotherm. Since the Δ*G*^o^_ads_ values were found to be between − 20.1 and − 26.1 kJ mol^−1^, the analyzed isotherm plots demonstrated that the adsorption process for these derivatives on CS surface is a mixed-type inhibitors. Scanning electron microscopy (SEM), energy dispersive X-ray spectroscopy (EDX), atomic force microscope (AFM) and Fourier- transform infrared spectroscopy (FTIR) were utilized to study the surface morphology, whereby, quantum chemical analysis can support the mechanism of inhibition. DFT data and experimental findings were found in consistent agreement.

**Graphical Abstract:**

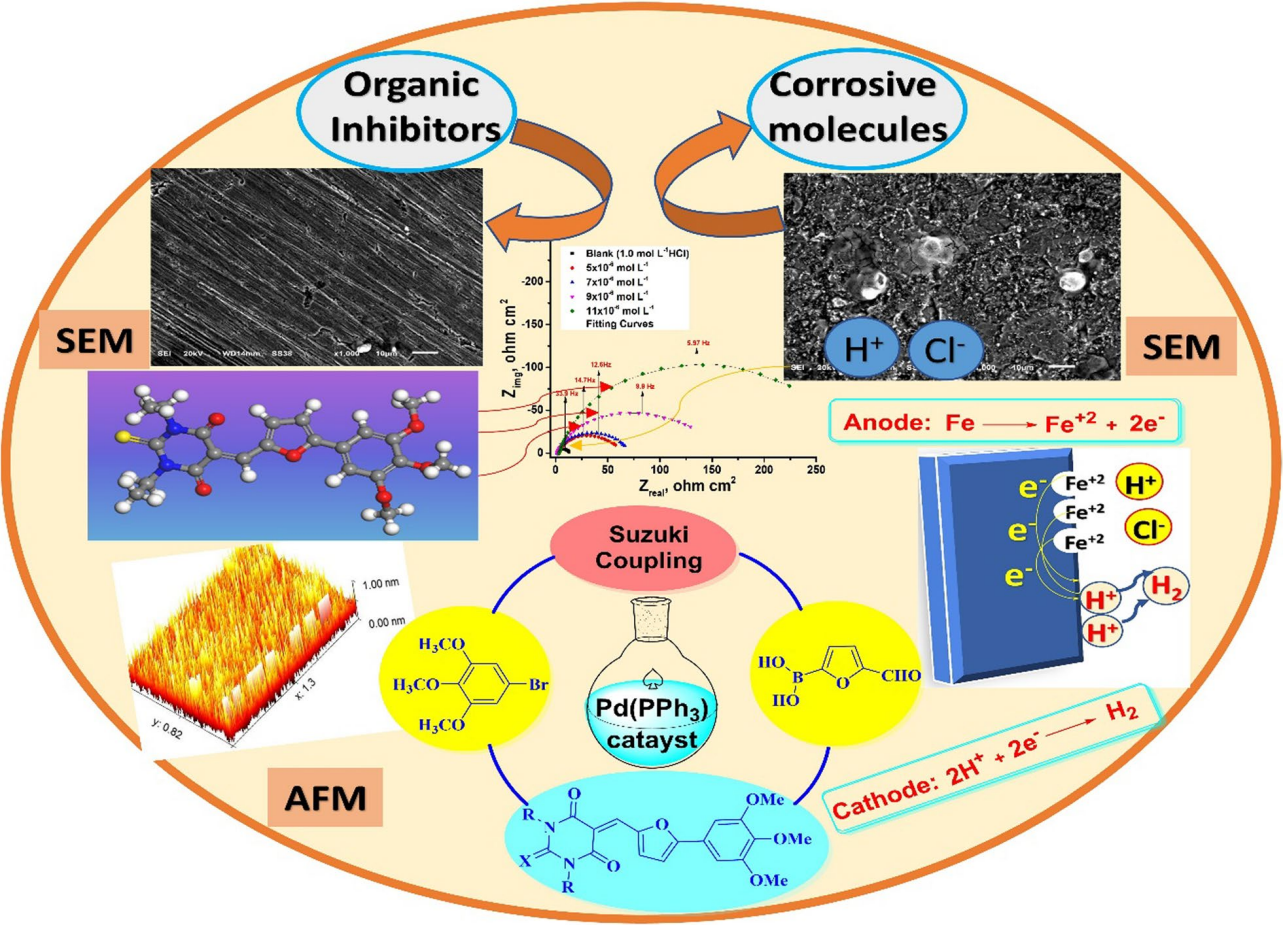

**Supplementary Information:**

The online version contains supplementary material available at 10.1186/s13065-024-01280-6.

## Introduction

Corrosion is a natural phenomenon [1–3] in which metals and alloys transform into more stable forms such as oxides and sulfides by reacting directly with the surrounding environment [4, 5]. However, some metallic components exposed to corrosive aqueous media, especially in acidic conditions, may suffer severe degradation of their properties and durability, leading to the disintegration of CS and failures [6]. Corrosion has significant implications for human safety [7] and various industries due to its negative impact, notably the gas and oil sector, making it a critical area of research [8]. Carbon steel (CS) is a vital component in construction and industrial field [9–12] due to its high mechanical properties, low temperature toughness, hydrogen-induced crack and fracture resistances, weldability [13] and remarkable economy, besides the possibilities for its environmental, technical and economic recycling in the concrete production industry [14–16]. However, one of the major drawbacks of using CS is its high susceptibility to corrosion in corrosive conditions, such as during the pickling process using HCl [17–19], which is widely employed in industries such as chemical cleaning [20], pickling iron, boiler descaling, scrubbing, [21, 22] and oil well acidification [23, 24]. Nevertheless, carbon steel corrosion is an inevitable but controllable phenomenon [22]. Among the available methods for corrosion control in acidic solutions, the use of inhibitors is considered an effective approach for protecting metals from corrosion [25]. Organic inhibitors containing π conjugated electrons, aromatic rings and heteroatoms are commonly used to prevent metal corrosion [26–29]. Organic scaffolds containing active sites such as oxygen, nitrogen and sulfur in their structures show higher inhibitory efficiency than other molecules having only a single heteroatom [30] through either chemical, physical or both adsorption mechanisms on the metal surface [31–33]. The aforementioned inhibitors block the active sites on the CS surface by forming protective coating layers [34] and reducing the corrosive effects [35]. The adsorption process can be influenced by the inhibitor structure [36], the nature of the metal surface, and the type of corrosive conditions [37]. Motivated by the above-mentioned aspects, the synthesis and investigation of new eco-friendly corrosion inhibitors is highly desirable, as the application of green chemistry is essential to the field of corrosion research. The percentages of inhibition efficiency (*η*) of some reported analogues of pyrimidine derivatives are shown in Table 1.

**Table 1:** Literature reviews on the corrosion inhibition behavior of similar pyrimidine derivatives studied before

Inhibitor	Corrosive medium	Conc. Of inhibitor	IE		Sample	Refs.
			PDP	EIS		
(*E*)-2-(Hexadecylthio)-8-(4- methoxybenzylidene)-5-(4-methoxyphenyl)-1,2,3,6,7,8-hexahydro-4H-cyclopenta[5, 6] pyrido[2,3-*d*] pyrimidin 4 -one (**Compound I**)	5% Sulfamic acid	1 × 10^–4^	82.1	84.4	X52 CS	[38]
(*E*)-8-(4-Methoxybenzylidene)-5-(4-methoxyphenyl) -2-thioxo1,2,3,6,7,8-hexahydro-4*H*-cyclopenta [5, 6] pyrido [2,3-*d*] pyrimidin-4-one (**Compound II**)			88.4	91.2		
5-(3,4 Dimethoxybenzylidene)-1,3-dimethylpyrimidine-2,4,6(1*H*,3*H*,5*H*)-trione (**I**)	1.0 M HCl	21 × 10^–6^	90.6	87.0	CS	[39]
5-(3,4 Dimethoxybenzylidene)-1,3-diethyl-2-thioxodihydropyrimidine-4,6(1*H*,5*H*)-dione (**II**)			92.4	91.3		
5-[4-(Dimethylamino) benzylidene]-1,3-dimethylbarbituric acid	1.0 M HCl	21 × 10^–6^	86.9	85.9	CS	[40]
4-(2-Fluorophenyl)-5-(ethoxycarbonyl)-6-methyl-3,4-dihydropyrimidin-2(1*H*)-one (2-FDHPM)	0.5 M H_2_SO_4_	1×10^–3^	86.246	79.7896	XC48 CS	[41]
4-(4-Fluorophenyl)-5-(ethoxycarbonyl)-6-methyl-3,4-dihydropyrimidin-2(1*H*)-one (4-FDHPM)			94.31	91.0208		
5-((4’-(Dimethylamino)-[1,1’-biphenyl]-4-yl)methylene)-1,3-diethyl-2-thioxodihydropyrimidine-4,6(1*H*,5*H*)-dione (HM-1228)	Oilfield produced water	0.5 mM	94.8	93.8	CS	[10]
5-((4’-(Dimethylamino)-[1,1’-biphenyl]-4-yl)methylene)-2-thioxodihydropyrimidine-4,6(1*H*,5*H*)-dione (HM-1227)			92.4	91.5		
5-((4’-(Dimethylamino)-[1,1’-biphenyl]-4-yl)methylene)pyrimidine-2,4,6(1*H*,3*H*,5*H*)-trione (HM-1226)			89.7	90.6		
5-((5-(3,4,5-Trimethoxyphenyl)furan-2-yl)methylene)pyrimidine-2,4,6(1*H*,3*H*,5*H*)-trione (HM-1221)	1.0 M HCl	11 × 10^−6^	87.8	89.3	CS	Our work
2-Thioxo-5-((5-(3,4,5-trimethoxyphenyl)furan-2-yl)methylene)dihydropyrimidine-4,6(1*H*,5*H*)-dione (HM-1222)			90.3	90.0		
1,3-Diethyl-2-thioxo-5-((5-(3,4,5-trimethoxyphenyl)furan-2-yl)methylene)dihydropyrimidine-4,6(1*H*,5*H*)-dione (HM-1223)			91.6	92.9		
1,3-Dimethyl-5-((5-(3,4,5-trimethoxyphenyl)furan-2-yl)methylene)pyrimidine-2,4,6(1*H*,3*H*,5*H*)-trione (HM-1224)			89.8	89.7		

The aim of the present work was to design and investigate new synthesized trimethoxyphenylfuran pyrimidinone derivatives as potential CS corrosion inhibitors at low concentrations in an acidic medium. This study involved electrochemical measurements, weight loss analysis, and surface examination of CS using AFM, SEM, FTIR and EDX methods. Moreover, the thermodynamic and kinetic parameters were calculated and discussed. The adsorption of four furan pyrimidinone scaffolds on the CS was also investigated at different soaking times to understand the interactions between the furylidene-pyrimidinone scaffolds and the CS surface. Furthermore, the proposed mechanism for corrosion inhibition was elucidated by quantum chemistry calculations for the four furylidene-pyrimidinone derivatives. Ultimately, we aimed to use these inhibitors to prevent pipeline corrosion and rusting in various industrial processes.

## Experimental

### Materials

Table 2 illustrates the molecular structures, formulas, molecular weights, yield, shape and melting point (m.p.) of four novel furylidene-pyrimidinone derivatives, **HM-1221**, **HM-1222**, **HM-1223**, and **HM-1224**. The synthesis and characterization in details are shown in the experimental section. (For IR, NMR, and Mass spectra of the investigated inhibitors see the supplementary material).

**Table 2:** The chemical and physical properties of investigated inhibitors

Inhibitor code	Molecular structures	Chemical properties
**HM-1221**		F.wt. = C_18_H_16_N_2_O_7_
		M.wt = 372.33
		Yield = 69%
		Shape: Red powder
		M.P. = 296–298
**HM-1222**		F.wt. = C_18_H_16_N_2_O_6_S
		M.wt = 388.39
		Yield = 78%
		Shape: Dark Red powder
		M.P. = 280–282
**HM-1223**		F.wt. = C_22_H_24_N_2_O_6_S
		M.wt = 444.50
		Yield = 73%
		Shape: Reddish brown powder
		M.P. = 178–179
**HM-1224**		F.wt. = C_20_H_20_N_2_O_7_
		M.wt = 400.39
		Yield = 71%
		Shape: Orange powder
		M.P. = 240–242

#### Methodology for synthesizing of the investigated inhibitors

##### Synthesis of furylidene- pyrimidinone derivatives 5a-d

5-(3,4,5-Trimethoxyphenyl) furan-2-carbaldehyde (**3**, HM-1220):

A mixture of 5-bromo-1,2,3-trimethoxybenzene **1** (2.50 g, 10 mmol), and Pd (PPh_3_)_4_ (250 mg) was dissolved in 20 mL toluene, then adding Na_2_CO_3_ (10 mL, 2M), and methanolic solution of (5-formylfuran-2-yl) boronic acid **2** (1.68g, 12 mmol). The mixture was allowed to heat at 80°C with stirring for ~ 12 h, after that extraction with ethyl acetate (250 mL, 3x). The resultant product was recrystallized from EtOH to yield 5-(3,4,5-trimethoxyphenyl) furan-2-carbaldehyde **3** as an anticipated product. Compound **3** was attained in 59% yield as a yellow solid, m.p. = 117–118°C, Lit [42] m.p. = 118 ℃. IR (KBr) ν^\^/cm^−1^: 3102 (sp^2^ C–H), 2932 (sp^3^ CH), 2844, 2805 (C–H of CHO), 1725, 1688, 1590 (C=O and C=C). MS (EI) m/e (rel.int.) for C_14_H_14_O_5_ (262.26); 262.97 (M^+^, 91.01%), 159.01 (100%).

##### General procedure for the synthesis of furylidene-pyrimidinone derivatives **5a-d**

Condensation reaction of a mixture of furan-2-carbaldehyde **3** (200 mg, 0.76 mmol), active methylene groups (1.52 mmol), namely, barbituric, thiobarbituric, 1,3-diethyl-2-thiobarbituric, 1,3-dimethylbarbituric acid in a mixture of 30 mL MeOH/AcOH (2:1) was refluxed for 12 h. While hot, the aforementioned products **5a-d** were obtained by filtering the precipitate, washing it with MeOH, and recrystallizing it from the suitable solvent.

5-((5-(3,4,5-Trimethoxyphenyl)furan-2-yl)methylene)pyrimidine-2,4,6(1*H*,3*H*,5*H*)-trione *(5a, HM-1221)*

IR (KBr) ν^\^/cm^−1^: 3388 (NH), 3189, 3120, 3053 (sp^2^ C–H), 2985, 2961, 2933 (sp^3^ C–H), 1751, 1661 (C=O), 1591, 1538, 1481 (C=C) (Figure S1). ^1^H-NMR (DMSO-*d*_*6*_); δ ppm 3.70 (s, 3H, *p*-methoxy-H’s), 3.87 (s, 6H, *m*-dimethoxy-H’s), 7.25 (s, 2 H_arom_), 7.48 (d, *J* = 3.5 *Hz*, 1H, furan-H), 8.13 (s, 1H, =CH), 8.58 (d, *J* = 3.5 *Hz*, 1H, furan-H), 11.23 (s, 1H exchangeable with D_2_O, NH), 11.32 (s, 1H exchangeable with D_2_O, NH) (Figure S2). ^13^C-NMR; δ ppm 56.18 (2C), 60.24, 102.84 (2C), 111.41, 111.69, 123.84, 129.79, 136.11, 139.33, 149.86, 150.31, 153.51 (2C), 160.45, 162.30, 163.57 (Figure S2). MS (EI) m/e (rel.int.) for C_18_H_16_N_2_O_7_ (372.33); 372.14 (M^+^, 29.18%), 226.98 (100%) (Figure S3).

2-Thioxo-5-((5-(3,4,5-trimethoxyphenyl)furan-2-yl)methylene)dihydropyrimidine-4,6(1*H*,5*H*)-*dione* *(5b, HM-1222)*

IR (KBr) ν^\^/cm^−1^: 3424 (N–H), 2925 (sp^3^ C–H), 1651 (C=O), 1541 (C = C), 1379 (C=S) (Figure S1). ^1^H-NMR (DMSO-*d*_6_);δ ppm 3.71 (s, 3H, *p*-methoxy-H’s), 3.88 (s, 6H, *m*-dimethoxy-H’s), 7.28 (s, 2H_arom_), 7.55 (d, *J* = 4.0 *Hz*, 1H, furan-H), 8.14 (s, 1H, =CH), 8.66 (d, *J* = 4.0 *Hz*, 1H, furan-H), 12.36 (s, 1H, NH), 12.41 (s, 1H, NH) (Figure S2). MS (EI) m/e (rel.int.) for C_18_H_16_N_2_O_6_S (388.39); 388.94 (M^+^, 100%) (Figure S3).

1,3-Diethyl-2-thioxo-5-((5-(3,4,5-trimethoxyphenyl)furan-2-yl)methylene)dihydropyrimidine-4,6(1*H*,5*H*)-dione *(5c, HM-1223):*

IR (KBr) ν^\^/cm^−1^: 3167, 3113 (sp^2^ C–H), 2977, 2932, 2833 (sp^3^ C–H), 1691, 1659 (C=O), 1566, 1477 (C=C), 1387 (C=S) (Figure S1). ^1^H-NMR (DMSO-*d*_6_); δ ppm 1.18–1.24 (m, 6H, CH_3_ of diethyl groups-H’s), 3.72 (s, 3H, *p*-methoxy-H’s), 3.89 (s, 6H, *m*-dimethoxy-H’s), 4.40–4.47 (m, 4H, CH_2_ of diethyl groups-H’s), 7.32 (s, 2H_arom_), 7.60 (d, *J* = 4.0 *Hz*, 1H, furan-H), 8.27 (s, 1H, =CH), 8.73 (d, *J* = 4.0 *Hz*, 1H, furan-H) (Figure S2). ^13^C-NMR; δ 12.16, 12.20, 42.77, 43.37, 56.20 (2C), 60.25, 103.26 (2C), 110.87, 112.61, 123.49, 131.84, 138.32, 139.83, 150.40, 153.53, 158.55 (2C), 160.47, 162.01, 178.47 ppm (Figure S2). MS (EI) m/e (rel.int.) for C_22_H_24_N_2_O_6_S (444.50); 444.99 (M^+^, 93.25%), 334.24 (100%) (Figure S3).

1,3-Dimethyl-5-((5-(3,4,5-trimethoxyphenyl)furan-2-yl)methylene)pyrimidine-2,4,6(1*H*,3*H*,5*H*)-trione (5d, HM-1224)

IR (KBr) ν^\^/cm^−1^: 3170, 3128 (sp^2^ C–H), 2942, 2839 (sp^3^ C–H), 1724, 1663 (C=O), 1576, 1476 (C=C) (Figure S1). ^1^H-NMR (DMSO-*d*_6_); δ ppm 3.24 (s, 6H, 2CH_3_), 3.72 (s, 3H, *p*-methoxy-H’s), 3.89 (s, 6H, *m*-dimethoxy-H’s), 7.29 (s, 2 H_arom_), 7.53 (d, *J* = 4.0 *Hz*, 1H, furan-H), 8.24 (s, 1H, =CH), 8.65 (d, *J* = 4.0 *Hz*, 1H, furan-H) (Figure S2). MS (EI) m/e (rel.int.) for C_20_H_20_N_2_O_7_ (400.39); 400.34 (M^+^, 65.08%), 274.96 (100%) (Figure S3).

#### Materials

Corrosion inhibition experiments have been carried out on CS with the following chemical composition (wt. %): (C: 0.07, Si: 0.05, Ti: 0.001, Mn: 0.3, Al: 0.03, S: 0.01, P: 0.022, and Fe balance). The materials were cut into coupons of size 2 cm × 2 cm × 0.2 cm for the WL tests. The working electrode used in surface morphology and electrochemical studies has an exposed area of 1 cm^2^. All the chemicals and reagents were purchased from Sigma-Aldrich Chemicals, all were of analytical grade, and solutions were prepared using double distilled water.

#### Solutions

One molar HCl (37%) stock solution was made by dilution with double-distilled water. The synthesized compounds were dispersed in a combination of 5 mL DMSO and 25 mL EtOH to generate a stock solution of dosage inhibitors with a concentration of 1 × 10^−3^ M. Furthermore, the concentration varieties of the studied compounds were (1 × 10^–6^ -11 × 10^–6^ M) and were prepared by dilution.

### Weight loss (WL) method

We measured WL using CS specimens at (30–50 °C) temperatures. Prior to being submerged in the test solution, the CS surface was polished using sandpaper grades (320–2000), cleaned with distilled water, allowed to dry at ambient temperature, and weighed. CS specimens were weighed before and after immersion in 100 mL of 1.0 M HCl without and with varied inhibitor dosages every 30 min for 3 h. The following equations were used to determine *CR*, *θ*, and *η* [43–46]:

1 $${C}_{R}=\frac{W}{At}$$

2 $$\theta = \frac{{C}_{\text{R}}-{C}_{R(i)}}{{C}_{R}}$$

3 $${\eta }_{\text{WL}}= \theta \times 100$$

whereas, W and A represent specimen WL (mg) and area (cm^2^), *CR* and *CR*_(i)_ represent CS corrosion rate (mg cm^−2^ h^−1^) without and with inhibitors, and *t* represents exposure duration (h), $\theta$ degree of surface coverage.

### Electrochemical measurements

In order to record and retain data, electrochemical procedures were done utilizing Potentiostat/Galvanostat (Gamry PCI300 /4) that include DC 105 software for PDP and EIS 300 programs for EIS measurements, is linked to a computer for data recording and storage. Electrochemical methods using EIS and PDP in 1.0 M HCl without and with varying inhibitor dosages at ambient temperature were used to study CS corrosion. The standard electrochemical cell has three glass vessels with a platinum wire (auxiliary electrode), a saturated calomel electrode, SCE, (reference electrode), and CS (working electrode). The exposed surface area of the working electrode was 1cm^2^. It was weld from one side to Cu-wire which used for electric connection. The samples were embedded in glass tube of just larger diameter than the samples then epoxy resin was used to stick the sample to glass tube. While, the chemical composition of the working electrode utilized in electrochemical methods was the same for the CS in weight loss. For 30 min, the CS electrode was submerged in the test solution to achieve a constant open circuit potential (OCP). Polarization studies were conducted in the potential range from − 250 mV to 250 mV vs. OCP above OCP at a scan rate of 0.5 mV s^−1^ scan rate. Corrosion current density (*i*_corr_) and corrosion potential (*E*_corr_) were assessed from the interplay of the correlation anodic and cathodic sections of Tafel plots in the presence and absence of altered inhibitor concentrations. EIS measurements were performed after immersing the electrode for 30 min, the EIS spectra were collected at the open circuit potential (OCP), the peak-to-peak voltage of the AC signal was 10 mV, and the resonant frequency evaluated was 0.01–10^5^ Hz. The important variables derived from the analysis of the Nyquist diagram are the resistance of charge transfer (*R*_ct_) and the capacity of the double layer (*C*_dl_).

### Surface analysis

#### SEM analysis

CS surface morphology and elemental composition were studied by scanning electron microscopy (SEM) Model (Quanta 250 FEG, originated in FEI Company in the Netherlands) with and without organic inhibitors.

#### AFM analysis

The micrographs and surface roughness of CS with and without the optimum concentration of organic inhibitors were investigated on the nanosurf C300 software of version 3.5.0.31 by employing AFM in contact FlexAFM3 mode with a nonconductive silicon probe.

### Quantum chemical calculations and Monte Carlo simulation studies:

Using the Material Studio D-MOL3 program, quantum chemical calculations were used to investigate the effectiveness of the trimethoxyphenylfurylidene-pyrimidinone derivatives' ability to suppress corrosion. Density Functional Theory (DFT) was utilized for the calculations, with the basis set DNP (4.4) function GGA. The COSMO solvation model was also employed. By using DFT, the quantum chemical parameters *E*_HOMO_, *E*_LUMO_, and ∆*E* were derived and examined. In order to identify the adsorption configurations of four investigated inhibitors on the interface of Fe (110), MC simulation was employed. Whereas, all computations were employed using the force field COMPASS (Condensed-Phase Optimized Molecular Potential for Atomistic Simulation Study).

## Results and discussion

### Synthesis and characterization of inhibitors

**Fig. 1:**
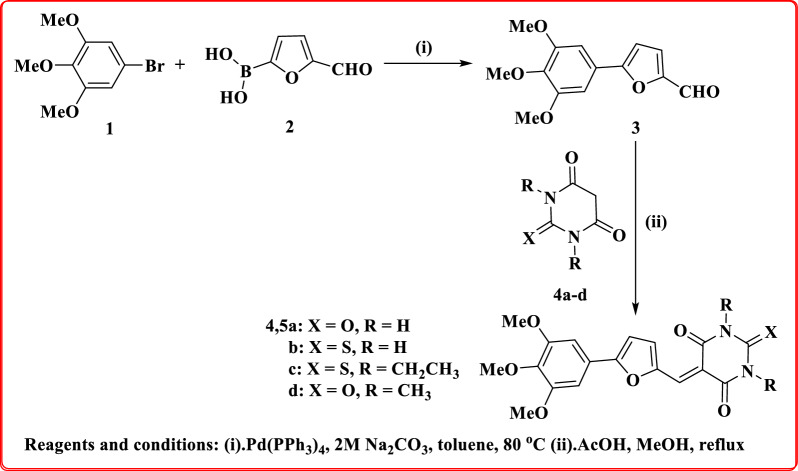
Synthesis of the new trimethoxyphenylfurylidene-pyrimidinones **5a-d**

The innovative trimethoxyphenylfurylidene-pyrimidinones **5a-d** were prepared starting with a Suzuki coupling reaction of bromo-trimethoxybenzene **1** with formylfuran-2-yl boronic acid **2** with the addition of Pd(0), Na_2_CO_3_ (2M) and heating at 80°C in a mixture of toluene and MeOH to afford furylcarbaldehyde **3**, after that compound **3** was condensed with barbituric acid (**4a**), 2-thiobarbituric acid (**4b**), 1,3-diethyl-2-thiobarbituric acid (**4c**), and 1,3-dimethyl barbituric acid (**4d**) to yield furylidene constitutions **5a-d** in acceptable yields (69–78%) as shown in Fig. 1.

Novel trimethoxyphenylfurylidene-pyrimidinones **5a-d** were synthesized and elucidated by spectral data. IR spectrum of compounds **5a-d** in the range of 1651 to 1751 cm^−1^, indicated the presence of carbonyl groups, while, compounds **5a** and **5b** showed bands at 3388 cm^−1^ (**5a**), 3424 cm^−1^ (**5b**) for NH, whereas thione groups were showed at 1379 cm^−1^ (**5b**), and 1387 cm^−1^ (**5c**). Whereas, ^1^H-NMR of hybrid **5a** displayed three singlet signals of 3,4,5-trimethoxyphenyl moiety at *δ* 3.70 (*para*-methoxy group, 3H), *δ* 3.87 (*meta*-dimethoxy groups, 6H), *δ* 7.25 (2H) corresponding to aromatic hydrogens, in addition to two doublet signals at *δ* 7.48 (1H) and 8.58 (1H) with coupling constant *J* = 3.5 *Hz* referring to 2,5-disubstituted furan moiety, one methylidene singlet signal at *δ* 8.13 (1H), plus two singlet signals of NH in pyrimidinone moiety exchangeable with D_2_O at *δ* 11.23 and 11.32 ppm. Whereby, ^13^C-NMR spectrum of compound **5a** displayed 15 carbon-signals of its carbon network with characteristic carbons at *δ* 56.18 (carbons of *meta*-dimethoxy groups), *δ* 60.24 (carbon of *para*-methoxy group), and *δ* 160.45, 162.30, and 163.57 (carbons of carbonyl groups). Mass spectrometry of compound **5a** gave a molecular ion peak (m/e) at 372.14 (M^+^, 29.18%). The structure of skeleton **5c** was confirmed via its ^1^HNMR spectrum displaying two multiplet signals at *δ* 1.18–1.24 integrated for six hydrogens (2CH_3_ of 1,3-diethyl groups) and four aliphatic hydrogens corresponding to two methylene groups of 1,3-diethyl groups at *δ* 4.40–4.47 ppm, in addition to two singlet signals related to *para*-methoxy group and *meta*-dimethoxy groups at *δ* 3.72, and *δ* 3.89 ppm, respectively, as well as singlet signal of two aromatic protons of 3,4,5-trimethoxyphenyl ring at *δ* 7.32, along with two doublet signals at *δ* 7.60 (1H) and 8.73 (1H) with coupling constant *J* = 4 *Hz* referring to 2,5-disubstituted furan ring and one singlet signal at *δ* 8.27 (methylidene, 1H). Whereas, ^13^C-NMR of scaffold **5c** showed 19 carbon-signals with the most characteristic carbons resonating at *δ* 12.16, 12.20, 42.77, 43.37 ppm related to four carbons of diethyl groups, *δ* 56.20 corresponding to two carbons of *meta*-dimethoxy groups, *δ* 60.25 referring to *para*-methoxy carbon, additionally, two carbonyl carbons at δ 160.47, 162.01 ppm, and one thione carbon at *δ* 178.47 ppm. The mass spectrometry of compound **5c** exhibited a molecular ion peak (m/e) at 444.99 corresponding to C_22_H_24_N_2_O_6_S.

### Corrosion measurements

#### WL method

**Fig. 2:**
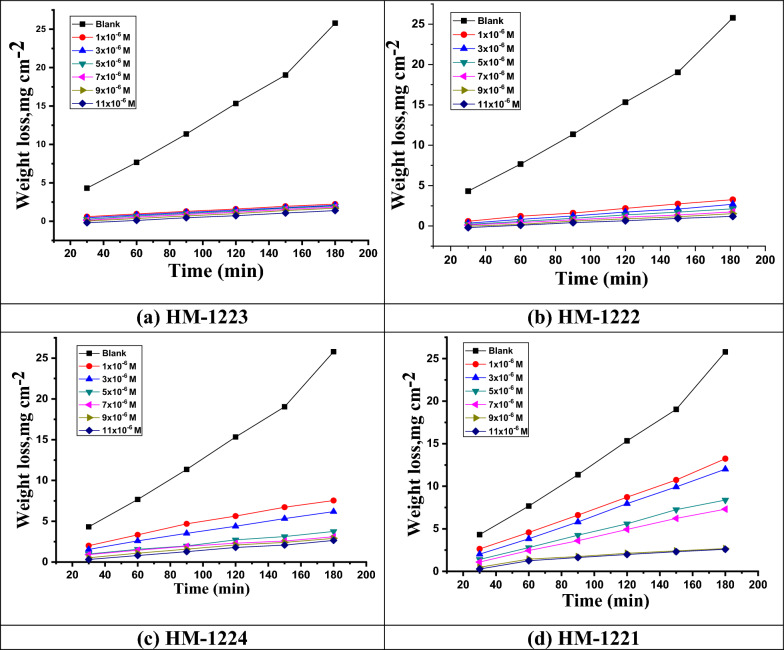
WL-time curve for CS in 1.0 M HCl with as well as without different concentrations of inhibitors **a-d** at 303 K

The WL method investigates the impact of dosage on the rate of corrosion of CS in 1.0 M HCl at different temperatures and well as lack diverse inhibitor doses (Fig. 2). The examination of the data in Table 3 displays that the *CR* of CS declines meaningfully and the *η* increases significantly with increasing the concentration dosages of inhibitors from 1 × 10^−6^ M to 11 × 10^−6^ M, this is due to the formation of a protective coating on the CS surface [47–49]. The inclusion of hetero nucleus atoms (N, O and S) in these tested molecules may be responsible for the efficacy of the inhibition process; as these atoms enhance the adsorption on CS via free electrons, which is crucial for the inhibition process [50].

**Table 3:** *WL* corrosion parameters of CS in 1.0 M HCl at various temperatures 303–323 K and with as well as lack various doses of inhibitors

Inhibitor	Conc., M × 10^–6^	*303 K*			308 K			*313 K*			318 K			*323 K*		
		*WL*	*CR*	*η*	*WL*	*CR*	*η*	*WL*	*CR*	*η*	*WL*	*CR*	*η*	*WL*	*CR*	*η*
–	Blank	*15.336*	*0.1278*	*–*	16.032	0.1326	–	*17.316*	*0.1443*	*–*	18.156	0.1513	–	*20.148*	*0.1679*	*–*
**HM-1223**	1	*1.733*	*0.0144*	*88.7*	2.485	0.0207	84.5	*3.532*	*0.0294*	*79.6*	6.427	0.0536	64.6	*7.294*	*0.0608*	*63.8*
	3	*1.641*	*0.0137*	*89.3*	2.277	0.0190	85.8	*3.255*	*0.0271*	*81.2*	4.811	0.0401	73.5	*6.286*	*0.0524*	*68.8*
	5	*1.518*	*0.0127*	*90.1*	2.036	0.0170	87.3	*2.805*	*0.0234*	*83.8*	3.395	0.0283	81.3	*5.682*	*0.0473*	*71.8*
	7	*1.304*	*0.0109*	*91.5*	1.731	0.0144	89.2	*2.320*	*0.0193*	*86.6*	3.304	0.0275	81.8	*4.674*	*0.0390*	*76.8*
	9	*1.242*	*0.0104*	*91.9*	1.395	0.0116	91.3	*1.766*	*0.0147*	*89.8*	3.032	0.0253	83.3	*4.070*	*0.0339*	*79.8*
	11	*0.843*	*0.0070*	*94.5*	1.283	0.0107	92.0	*1.489*	*0.0124*	*91.4*	2.033	0.0169	88.8	*3.083*	*0.0257*	*84.7*
**HM-1222**	1	*2.668*	*0.0222*	*82.6*	3.222	0.0269	79.9	*3.723*	*0.0310*	*78.5*	6.627	0.0552	63.5	*7.455*	*0.0621*	*63.0*
	3	*2.040*	*0.0170*	*86.7*	2.677	0.0223	83.3	*3.377*	*0.0281*	*80.5*	4.975	0.0415	72.6	*6.407*	*0.0534*	*68.2*
	5	*1.457*	*0.0121*	*90.5*	2.196	0.0183	86.3	*2.788*	*0.0232*	*83.9*	3.450	0.0287	81.0	*5.742*	*0.0479*	*71.5*
	7	*1.212*	*0.0101*	*92.1*	1.780	0.0148	88.9	*2.372*	*0.0198*	*86.3*	3.341	0.0278	81.6	*4.836*	*0.0403*	*76.0*
	9	*1.258*	*0.0105*	*91.8*	1.475	0.0123	90.8	*1.853*	*0.0154*	*89.3*	3.195	0.0266	82.4	*4.110*	*0.0343*	*79.6*
	11	*1.012*	*0.0084*	*93.4*	1.347	0.0112	91.6	*1.645*	*0.0137*	*90.5*	2.124	0.0177	88.3	*3.365*	*0.0280*	*83.3*
**HM-1224**	1	*5.828*	*0.0486*	*62.0*	5.980	0.0498	62.7	*6.372*	*0.0531*	*63.2*	6.809	0.0567	62.5	*8.099*	*0.0675*	*59.8*
	3	*4.723*	*0.0394*	*69.2*	4.922	0.0410	69.3	*5.576*	*0.0465*	*67.8*	6.082	0.0507	66.5	*7.938*	*0.0662*	*60.6*
	5	*2.929*	*0.0244*	*80.9*	4.345	0.0362	72.9	*3.775*	*0.0315*	*78.2*	4.811	0.0401	73.5	*7.314*	*0.0609*	*63.7*
	7	*2.515*	*0.0210*	*83.6*	3.703	0.0309	76.9	*3.515*	*0.0293*	*79.7*	4.485	0.0374	75.3	*6.044*	*0.0504*	*70.0*
	9	*2.500*	*0.0208*	*83.7*	2.677	0.0223	83.3	*2.909*	*0.0242*	*83.2*	3.032	0.0253	83.3	*5.420*	*0.0452*	*73.1*
	11	*1.978*	*0.0165*	*87.1*	2.116	0.0176	86.8	*2.805*	*0.0234*	*83.8*	2.996	0.0250	83.5	*4.493*	*0.0374*	*77.7*
**HM-1221**	1	*8.803*	*0.0734*	*42.6*	9.587	0.0799	40.2	*10.563*	*0.0880*	*39.0*	11.329	0.0944	37.6	*13.016*	*0.1085*	*35.4*
	3	*8.450*	*0.0704*	*44.9*	8.914	0.0743	44.4	*9.766*	*0.0814*	*43.6*	10.422	0.0868	42.6	*11.726*	*0.0977*	*41.8*
	5	*5.736*	*0.0478*	*62.6*	8.000	0.0667	50.1	*8.797*	*0.0733*	*49.2*	9.296	0.0775	48.8	*10.396*	*0.0866*	*48.4*
	7	*5.122*	*0.0427*	*66.6*	6.926	0.0577	56.8	*7.532*	*0.0628*	*56.5*	8.679	0.0723	52.2	*9.913*	*0.0826*	*50.8*
	9	*2.316*	*0.0193*	*84.9*	5.852	0.0488	63.5	*7.394*	*0.0616*	*57.3*	8.443	0.0704	53.5	*9.550*	*0.0796*	*52.6*
	11	*2.178*	*0.0181*	*85.8*	4.104	0.0342	74.4	*5.749*	*0.0479*	*66.8*	6.191	0.0516	65.9	*8.059*	*0.0672*	*60.0*

#### Effect of temperature

**Fig. 3:**
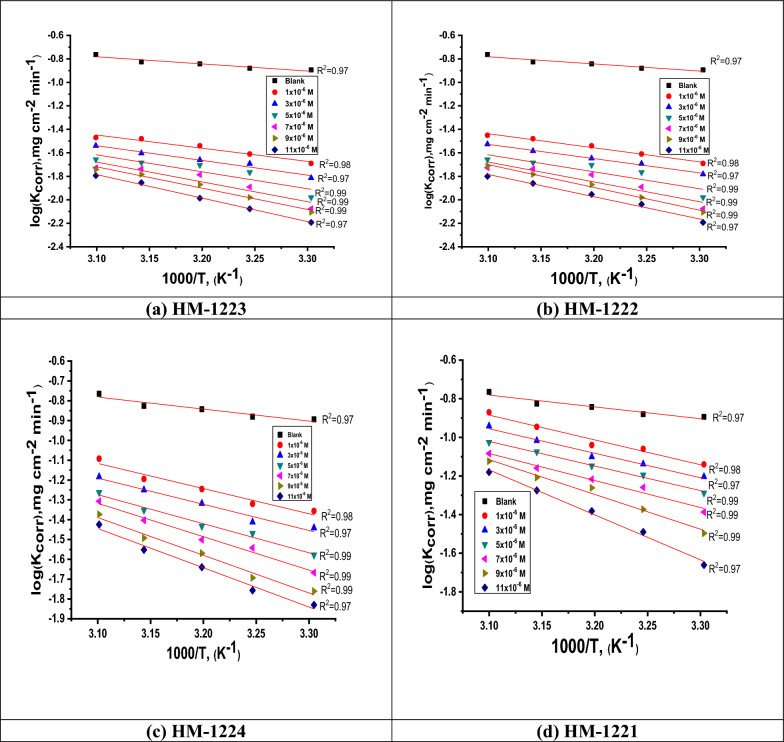
Arrhenius plots for CS corrosion in the 1.0 M HCl without as well as after using various concentrations of **a**–**d**

**Fig. 4:**
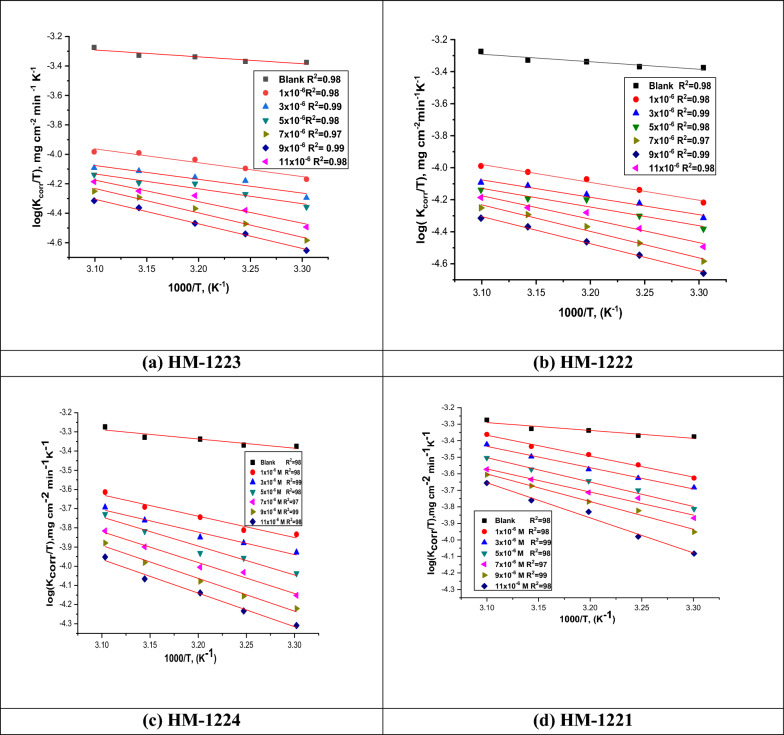
Kinetic transition state plots for CS dissolution in 1.0 M HCl without as well as after utilizing various doses of inhibitors **a**–**d**

During a three-hour immersion, the *WL* method was used to examine the impact of temperature on the percentage *η* at various temperatures ranging from 303 to 323 K, both with and without different dosages of organic inhibitors. While, Table 3 illustrates the decreasing values of the inhibition efficacy and increasing of the corrosion rate along with increasing temperature. This occurs as a result of the dissociation between inhibitor molecules and the metal surface. Apparently, the obtained results confirmed the inhibitor molecules blocking active sites by adsorption on the CS surface. Activation thermodynamic parameters were evaluated using the Arrhenius and transition state Eqs. [51–54]:

4 $$\text{log }{k}_{\text{corr}}=\left(\frac{-{E}_{\text{a}}^{*}}{2.303RT}\right)+\text{ log }A$$

5 $${k}_{\text{corr}}=\left(\frac{RT}{Nh}\right)exp\left(\frac{\Delta {S}^{*}}{R}\right)exp\left(\frac{-\Delta {H}^{*}}{RT}\right)$$

where *k*_corr_ represents the corrosion rate resulted from WL measurements, *R* denotes the gas constant, *T* represents the absolute temperature, *E*_a_^*^ signifies the apparent activation energy and *A* indicates the Arrhenius frequency factor, *N* refers to Avogadro's number, ∆H^*^ and ∆S^*^ embodies the enthalpy and entropy of activation, whereby *h* represents Planck's constant. While, the relation between Arrhenius plots of (log *k*_corr_) vs. (1000/*T*) for corrosion of metal in acidic medium of different doses of the inhibitors at diverse temperatures (30 − 50 °C) was represented in Fig. 3, and the straight lines were gotten with the slope (− *E*_a_^*^/2.303*R*) as well as intercept of log *A*. In the same context, the higher values of *E*_a_^*^ in the presence of inhibitors is attributed to that the physisorption mechanism [54] as shown in Table 4. Studying graphs of the transition state of (log *k*_corr_ /*T*) vs. (1000/*T*) for the inhibitors are presented in Fig. 4. The straight lines with a slope =  − ΔH^*^/*R* were achieved using ΔH^*^ and ΔS^*^ values. Whereas, a positive value for ΔH* suggests that the manufacturing of an activated complex is endothermic [55, 56] as shown in Table 4, whereas a negative value for ΔS* refers to the order is determined by the transformation of reactants into an activated complex [57, 58]. It is evident that for the inhibited solution the ΔS^*^ values are less negative compared to the uninhibited, as the rational probability attributed to desorption of H_2_O from the CS surface.

**Table 4:** Activation parameters gained from *WL* approach

Inhibitor	Conc. X10^−6^ M	-*E*_a_^*^	∆H^*^	− ∆S^*^
		kJ mol^−1^	kJ mol^−1^	J mol^−1^ K^−1^
	1.0 M HCl	13.4 ± 0.2028	10.85 ± 0.1732	228.4 ± 0.2214
**HM-1223**	1	33.6 ± 0.2211	31.05 ± 0.1232	215.6 ± 0.2001
	3	38.4 ± 0.2403	35.85 ± 0.1121	211.9 ± 0.2021
	5	38.2 ± 0.223	35.65 ± 0.1214	195.4 ± 0.2214
	7	39.3 ± 0.2021	36.75 ± 0.1120	191.3 ± 0.2021
	9	41.1 ± 0.1975	38.55 ± 0.1201	180.2 ± 0.1745
	11	44 ± 0.1754	41.45 ± 0.1214	157.8 ± 0.2141
**HM-1222**	1	23.4 ± 0.1245	20.85 ± 0.1724	214.4 ± 0.1245
	3	25.8 ± 0.1123	23.25 ± 0.1654	207.9 ± 0.2021
	5	29.5 ± 0.1745	26.95 ± 0.1714	194.6 ± 0.2144
	7	34.2 ± 0.2021	31.65 ± 0.1454	187.5 ± 0.1754
	9	39 ± 0.2214	36.45 ± 0.1987	177.2 ± 0.2144
	11	49.1 ± 0.1245	46.55 ± 0.1541	153.1 ± 0.2214
**HM-1224**	1	26 ± 0.1745	23.45 ± 0.1232	197.9 ± 0.2214
	3	27.2 ± 0.1245	24.65 ± 0.2145	195.7 ± 0.2211
	5	29.9 ± 0.1232	27.35 ± 0.2104	176.4 ± 0.1745
	7	34.1 ± 0.2541	31.55 ± 0.2001	172 ± 0.1245
	9	38.3 ± 0.1457	35.75 ± 0.1245	167.4 ± 0.1452
	11	39.8 ± 0.1245	37.25 ± 0.1932	146.4 ± 0.1745
**HM-1221**	1	26.2 ± 0.1952	23.65 ± 0.1564	183.9 ± 0.1657
	3	26.3 ± 0.1245	23.75 ± 0.1717	183.8 ± 0.2241
	5	26.5 ± 0.1423	23.95 ± 0.1254	173.9 ± 0.1245
	7	28.6 ± 0.1245	26.05 ± 0.1844	180 ± 0.1475
	9	36.3 ± 0.1751	33.75 ± 0.1932	164.1 ± 0.1745
	11	38.4 ± 0.1654	35.85 ± 0.1547	138.6 ± 0.1245

#### Study the adsorption isotherm

On the basis of mechanism of corrosion^'^, it is essential to understand how the inhibitors adsorb on the CS surface. The adsorption process could be explained as a substitution process between the organic molecules in the aqueous phase (Org_(sol)_) and H_2_O molecules previously adsorbed on the metal surface (H_2_O_(ads)_), the adsorption mechanism is accomplished according to the following equation.

**Fig. 5:**
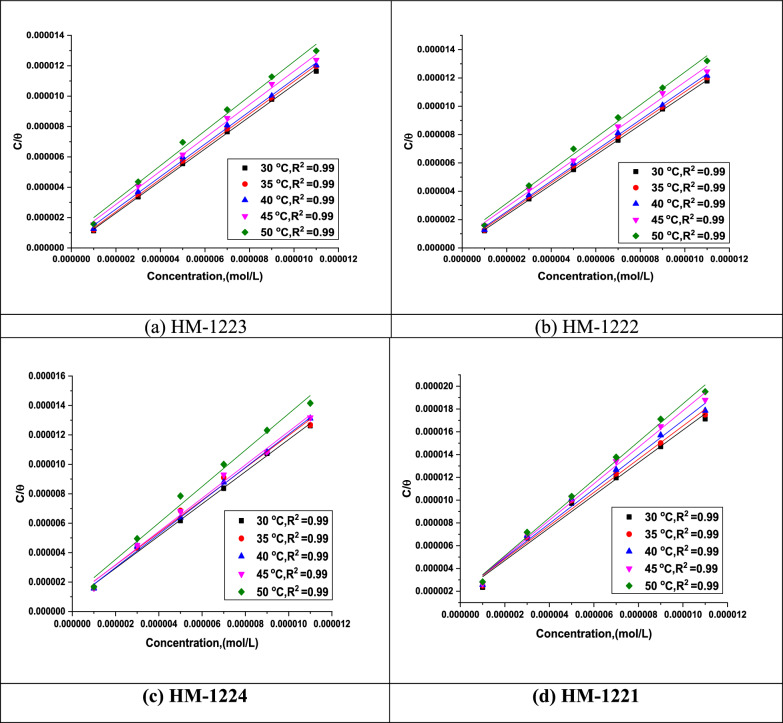
The plots of Langmuir isotherm for CS in 1.0 M HCl with altered doses of inhibitors **a-d** at diverse temperatures

**Fig. 6:**
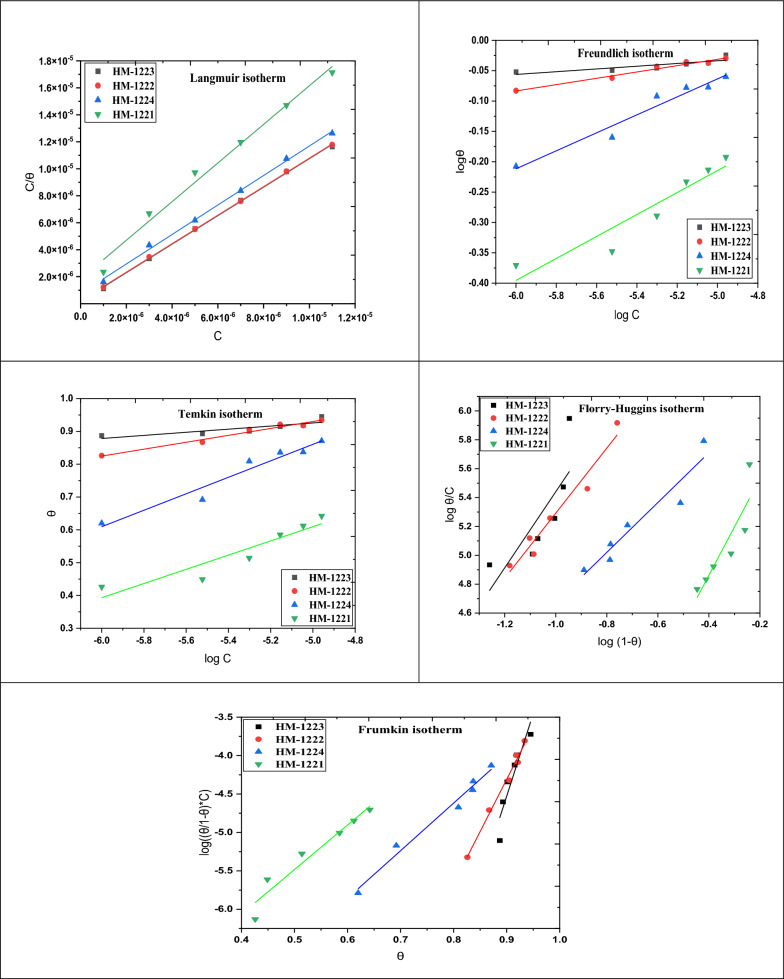
Various adsorption isotherms of the tested inhibitors for the corrosion of CS in 1.0 M HCl at 303 K

It is essential to understand how the inhibitors adsorb on the CS surface. As the reaction between organic hybrids in the aqueous phase (Org_aq_) and the H_2_O molecules underwent a similar manner to this adsorption according to following equation [59]:

6 $${\text{Org}}_{{({\text{sol}})}} + {\text{ x H}}_{{2}} {\text{O}}_{{({\text{ads}})}} \to {\text{ Org}}_{{({\text{ads}})}} + {\text{ x H}}_{{2}} {\text{O}}_{{({\text{sol}})}}$$

where x refers to the quantity of H_2_O molecules that the inhibitory molecules have displaced. Adsorption isotherms are helpful for investigating the interaction between the inhibitor molecules and the metal surface. Different isotherms, involving Langmuir, Frumkin, Temkin, Florry-Huggins and Freundlich were performed to determine the adsorption type that corresponded to the tested inhibitors. It is an evident that the correlation of the Langmuir isotherm is almost equal to unity (Fig. 5) shows that the Langmuir adsorption isotherm is obeyed when inhibitors are adsorbed on metal surfaces. Additional adsorption isotherms are discussed in Table 5 and showed in Fig. 6. The following Eq. was used to obtain the Langmuir adsorption isotherm [60, 61]:

7 $$\frac{{C}_{inh}}{\theta }=\frac{1}{{K}_{ads}}+ {C}_{inh}$$

whereas, the defined symbols in Eq. 7 are adsorption equilibrium constant (*K*_ads_), and the corrosion inhibitor dose in the solution (*C*_inh_). This equation was used to calculate the value of standard free energy of adsorption (∆*G*°_ads_) associated with *K*_ads_ for understanding of the inhibitors' adsorption process and their types [59, 60]:

8 $$K\text{ads}=\left(\frac{1}{55.5}\right)\text{ exp}\left(\frac{{-\Delta G}_{\text{ads}}^{^\circ }}{RT}\right)$$

whereas, *T* is the thermodynamic temperature (K), *R* is the universal gas constant, the molar concentration of water is 55.5. In addition, *K*_ads_ values are moderately high, indicating a strong inhibitors adsorption on CS [62] as illustrated in Table 6. Also, the highly negative value of ∆*G*°_ads_ demonstrates the adsorption occurs spontaneously [63]. According to the literature, if Δ*G*°_ads_ values at around (− 20 kJ mol^−1^) or lower negative, the adsorption of an inhibitor is a physisorption. In contrast, if the values of Δ*G*°_ads_ are (− 40 kJ mol^−1^) or higher negative is defined as chemisorption [64, 65]. From Table 6, the ∆*G*°_ads_ values of the synthesized scaffolds are round − 26 to − 20 kJ mol^−1^, indicating clearly that the mechanism is physisorption forming strong bonds. The Van't Hoff equation is used to calculate the heat of adsorption (Δ*H*°_ads_) (Eq. 9) [66]:

**Table 5:** Different adsorption isotherms of the tested inhibitors for the corrosion of CS in 1.0 M HCl at 303K

Adsorption isotherms	Inhibitors	Adsorption parameters		
		Regression coefficient (*R*^2^)	Slope	Intercept
Langmuir	**HM-1223**	0.99921	1.05666	1.80026E-7
	**HM-1222**	0.99975	1.05629	2.25186E-7
	**HM-1224**	0.99766	1.09323	7.5455E-7
	**HM-1221**	0.98697	1.43206	1.83385E-6
Freundlich	**HM-1223**	0.74772	0.02257	0.07928
	**HM-1222**	0.97525	0.05206	0.22904
	**HM-1224**	0.9549	0.1481	0.67703
	**HM-1221**	0.89234	0.18098	0.6904
Temkin	**HM-1223**	0.74011	0.04734	1.16237
	**HM-1222**	0.97434	0.10525	1.45618
	**HM-1224**	0.95025	0.25099	2.1154
	**HM-1221**	0.87266	0.21648	1.69195
Florry-Huggins	**HM-1223**	0.63907	2.63443	8.07479
	**HM-1222**	0.95093	2.25218	7.54432
	**HM-1224**	0.91132	1.72566	6.40104
	**HM-1221**	0.79081	3.34389	6.20012
Frumkin	**HM-1223**	0.85691	21.46338	-23.84599
	**HM-1222**	0.98763	13.57088	-16.52273
	**HM-1224**	0.98067	6.19138	-9.57126
	**HM-1221**	0.93442	5.79804	-8.38211

**Table 6:** The results of adsorption thermodynamic of organic scaffolds on CS in 1.0 M HCl at 303- 323 K

Inhibitors	Temp K	*K*_ads_ M^−1^	− Δ*G*°_ads_ kJ mole^−1^	-Δ*H*°_ads_ kJ mole^−1^	-Δ*S*°_ads_ J mole^−1^ k^−1^
**HM-1223**	303	5.55E + 02	26.1 ± 0.1854	67.3 ± 0.2142	136.149 ± 0.1214
	308	4.10E + 02	25.6 ± 0.1565		135.061 ± 0.1254
	313	2.41E + 02	24.7 ± 0.1254		135.99 ± 0.1412
	318	1.48E + 02	23.8 ± 0.2124		136.664 ± 0.1214
	323	1.17E + 02	23.5 ± 0.2144		135.342 ± 0.1754
**HM-1222**	303	4.44E + 02	25.5 ± 0.1524	54.9 ± 0.1624	97.126 ± 0.1214
	308	3.04E + 02	24.9 ± 0.1247		97.3341 ± 0.1234
	313	2.48E + 02	24.8 ± 0.1124		96.1793 ± 0.1425
	318	1.45E + 02	23.7 ± 0.1124		97.884 ± 0.1652
	323	1.19E + 02	23.6 ± 0.1414		96.8543 ± 0.1722
**HM-1224**	303	1.33E + 02	22.4 ± 0.1474	30.1 ± 0.1244	25.4227 ± 0.1241
	308	1.08E + 02	22.2 ± 0.1452		25.5391 ± 0.1785
	313	8.79E + 01	22.1 ± 0.1147		25.688 ± 0.1652
	318	7.54E + 01	22.0 ± 0.1457		25.4493 ± 0.1254
	323	6.31E + 01	21.9 ± 0.1425		25.4628 ± 0.1325
**HM-1221**	303	7.78E + 01	21.1 ± 0.1752	27.3 ± 0.1412	20.3729 ± 0.1574
	308	5.99E + 01	20.8 ± 0.1365		21.0864 ± 0.1475
	313	4.86E + 01	20.5 ± 0.1245		21.4108 ± 0.1547
	318	4.17E + 01	20.4 ± 0.1514		21.3145 ± 0.1874
	323	3.19E + 01	± 0.1321		22.2151 ± 0.1958

9 $${\text{log }K}_{\text{ads}}=\frac{-\Delta {H}_{\text{ads}}^{^\circ }}{2.303RT}+constant$$

**Fig. 7:**
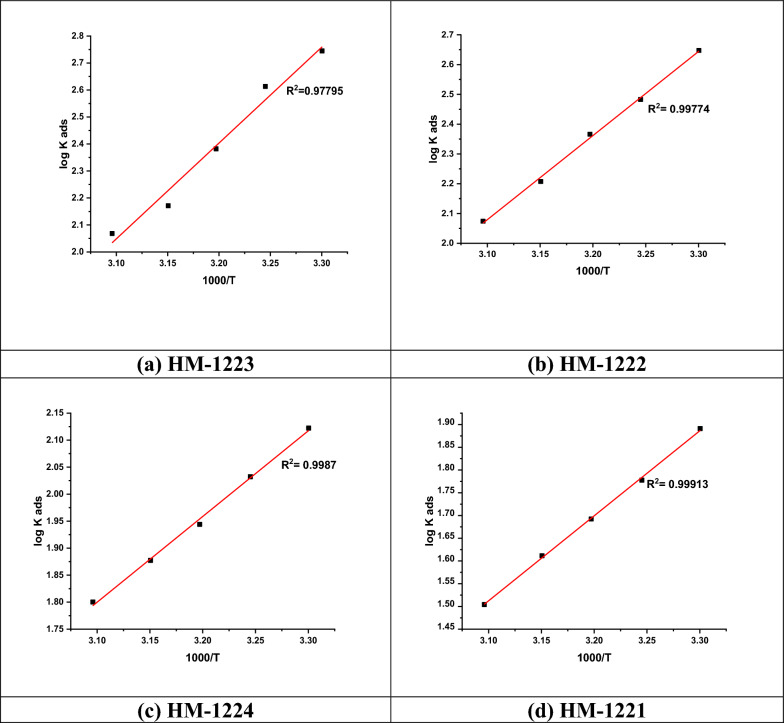
Vant's Hoff plots (Log *K*_ads_ vs. 1000/*T*) for the adsorption of organic molecules **a**–**d** at 303 K on CS surface in 1.0 M HCl

Figure 7 revealed the plots of Log (*K*_ads_) vs. 1000/*T* for inhibitors. Whereas, straight lines were attained with a slope =  − ∆*H*°_ads_/2.303*R* in which enthalpy were computed from and intercept =  − ∆*S*°_ads_/2.303*R* − log (55.5). Gibbs–Helmholtz equation is used to determine the standard adsorption entropy (ΔS°_ads_) at diverse temperatures [66]:

10 $${{\Delta S}^{^\circ }}_{\text{ads}}=\frac{\Delta {H}_{\text{ads}}^{^\circ }- \Delta {G}_{\text{ads}}^{^\circ }}{T}$$

Table 6 lists the values of *K*_ads_, △*G*°_ads_, enthalpy of adsorption (∆*H*°_ads)_, and the standard entropy (Δ*S*°_ads_). Whereas, the Δ*H*°_ads_ values are negative proving that the adsorption process is exothermic reaction [67], and the negative values of Δ*S*°_ads_ result from substitution process can be assigned to rising of entropy at the metal/solution interface due to replacing of the water molecules by inhibitor molecules in the solution [68].

### Electrochemical technique

#### Measurements of OCP

**Fig. 8:**
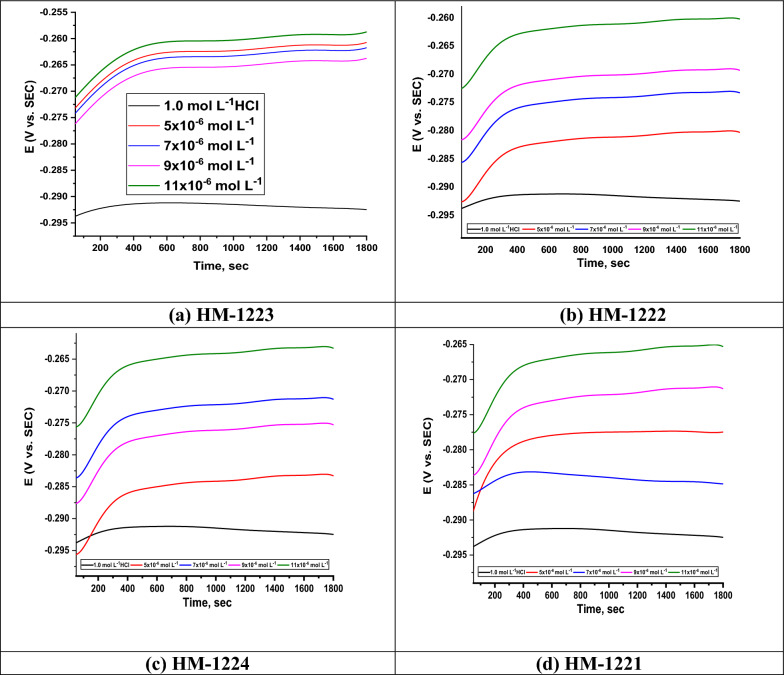
Changes in E_OCP_ vs. time for CS in the 1.0 M HCl either alone or with various dosages of organic hybrids **a**–**d** at 298 K

Figure 8 displays the relation of the OCP vs. time curves for CS in 1.0 M HCl in the absence besides utilizing varied concentrations of investigated compounds, (a) **HM-1223** (b) **HM-1222** (c) **HM-1224** (d) **HM-1221**, at 298 K. As the deterioration of the CS with corrosive layers on its surface was developed due to the fact of dissolution of the oxide film on the metal surface. From the OCP curves, it is noted that the potentials of inhibited solutions moved to more positive values contrasted to the uninhibited.

#### PDP technique

**Fig. 9:**
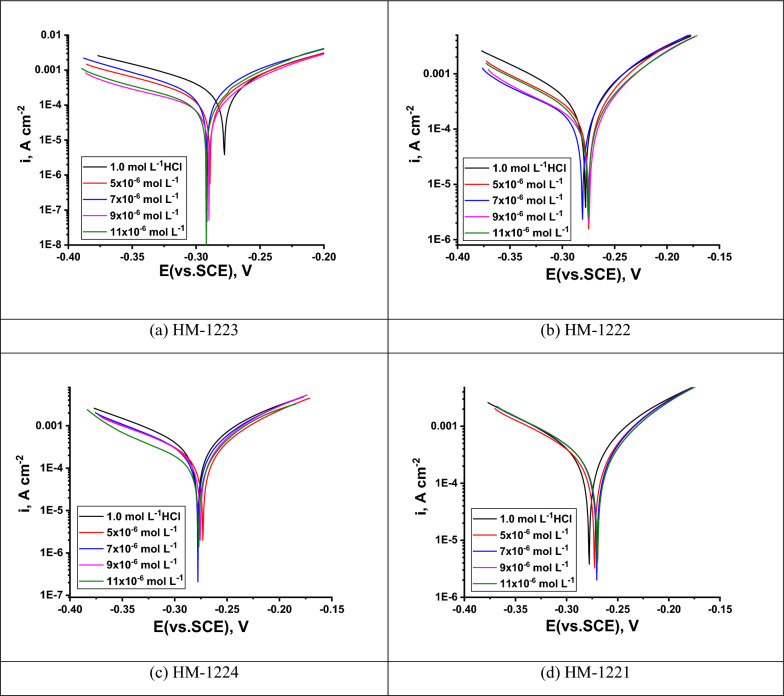
PDP curves for the CS corrosion in 1.0 M HCl at 298 K without and after adding diverse concentrations of inhibitors **a**–**d**

Polarization measurements were performed for investigation the kinetics of cathodic and anodic reactions. As indicated in Fig. 9, it is clear that the presence of inhibitors causes a marked decrease in the corrosion rate. The inhibitors have a significant effect on the rate of the hydrogen evolution and anodic dissolution reactions i.e. the investigated inhibitors act as mixed type inhibitors. the extrapolation of the polarization curves yields the electrochemical corrosion parameters like (*i*_corr_, *E*_corr_, *β*_a_, *β*_c_ and *η*) which are reported in Table 7. Also, *i*_corr_ values are utilized to calculate *η* (Eq. 11) [69]:

11 $${\eta }_{\text{PDP}}=\left(\frac{{{i}_{\text{corr}} - i}_{\text{corr }(\text{inh})}}{{i}_{\text{corr}}}\right) \times 100$$

Where as, *i*_corr_ and *i*_corr (inh.)_ refer to the corrosion current densities in acidic solution in the absence in addition to existence of organic molecules, respectively, while (*β*_a_), (*β*_c_) and *E*_corr_ represent anodic, cathodic Tafel and the corrosion potential. Table 7 demonstrates that the corrosion current density dropped when the inhibitors were added and ${\eta }_{\text{PDP}}$ increases with increasing inhibitor concentrations. This was because the inhibitors are adsorbed onto the CS surface, reducing the rate of dissolution reaction by blocking active sites on the surface [70]. From the measurements, it was found that the corrosion potential gap is lower than 85 mV for all concentrations, and the anodic and cathodic partial currents are also decreased. The change in the *E*_corr_ value is (23 mV), these findings reveal the mixed character of the inhibitors under research [71, 72] and they also suggest that the inhibitors utilized diminish the anodic dissolving rates of CS and the reduction of H^+^. Both cathodic (*β*_c_) and anodic (*β*_a_) Tafel slopes do not change remarkably, which indicates that the mechanism of corrosion reaction does not change and the corrosion reaction is inhibited by blockage of active sites by the investigated inhibitors by simple adsorption mode [73]. % ${\eta }_{\text{PDP}}$ of these derivatives follows the sequence: **HM-1223** > **HM-1222** > **HM-1224** > **HM-1221**. The results acquired from the PDP measurements are closely matched with the outcomes of WL approach.

**Table 7:** PDP corrosion parameters of CS utilizing 1.0 M HCl without and with besides utilizing diverse doses of the organic constitutions **a-d** at 298 K

Inhibitor	Conc., M	*− E*_corr_ (mVvs.SCE),	*β*_a_, mV decˉ^1^	*− β*_c_, mV decˉ^1^	*i*_corr_, μA mˉ^2^	*θ*	*η*_PDP_
**HM-1223**	1.0 mol Lˉ^1^ HCl	278 ± 0.2011	88.5	176	990 ± 0.1754	–	–
	5 × 10ˉ^6^	289 ± 0.2144	80	227	137 ± 0.1711	0.862	86.2
	7 × 10ˉ^6^	291 ± 0.2214	72	179	128 ± 0.1952	0.871	87.1
	9 × 10ˉ^6^	289 ± 0.1952	54	251	95 ± 0.2145	0.904	90.4
	11 × 10ˉ^6^	292 ± 0.1722	54	260	83 ± 0.2111	0.916	91.6
**HM-1222**	5 × 10ˉ^6^	275 ± 0.1625	66	225	161 ± 0.2145	0.837	83.7
	7 × 10ˉ^6^	280 ± 0.1754	58	267	144 ± 0.1234	0.855	85.5
	9 × 10ˉ^6^	274 ± 0.1952	51	153	110 ± 0.2156	0.889	88.9
	11 × 10ˉ^6^	275 ± 0.1854	64	132	96 ± 0.2011	0.903	90.3
**HM-1224**	5 × 10ˉ^6^	273 ± 0.1625	66	139	188 ± 0.2014	0.810	81.0
	7 × 10ˉ^6^	277 ± 0.1524	65	202	157 ± 0.1245	0.841	84.1
	9 × 10ˉ^6^	277 ± 0.1754	66	135	125 ± 0.1857	0.874	87.4
	11 × 10ˉ^6^	275 ± 0.1874	63	142	101 ± 0.1574	0.898	89.8
**HM-1221**	5 × 10ˉ^6^	272 ± 0.1957	70	146	195 ± 0.1541	0.803	80.3
	7 × 10ˉ^6^	287 ± 0.1875	66	152	177 ± 0.1475	0.821	82.1
	9 × 10ˉ^6^	270 ± 0.1789	71	136	138 ± 0.1524	0.861	86.1
	11 × 10ˉ^6^	269 ± 0.1321	74	129	121 ± 0.1325	0.878	87.8

#### EIS technique

**Fig. 10:**
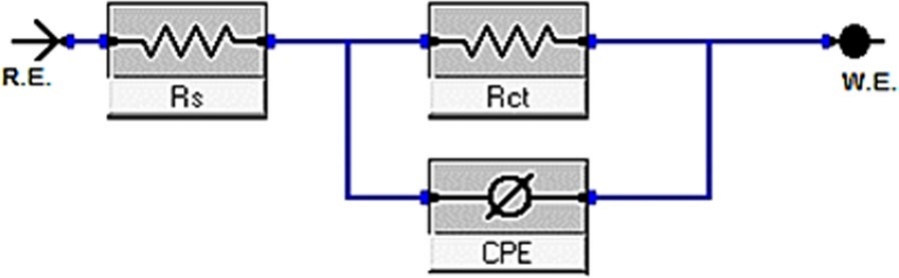
A simple circuit used to suit the EIS results

**Fig. 11:**
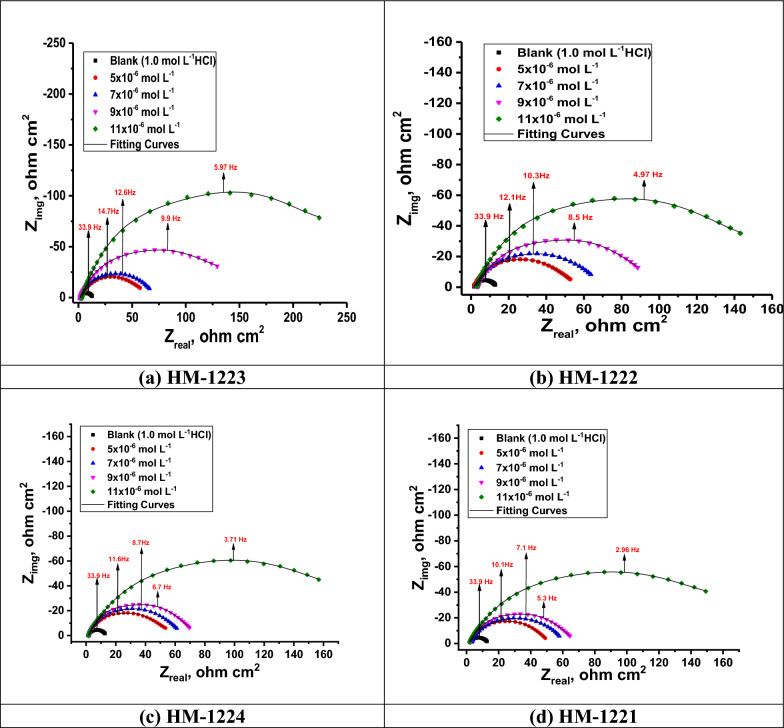
Nyquist plot for CS in 1.0 M HCl and with several doses of the inhibitors **a-d** at 298 K

**Fig. 12:**
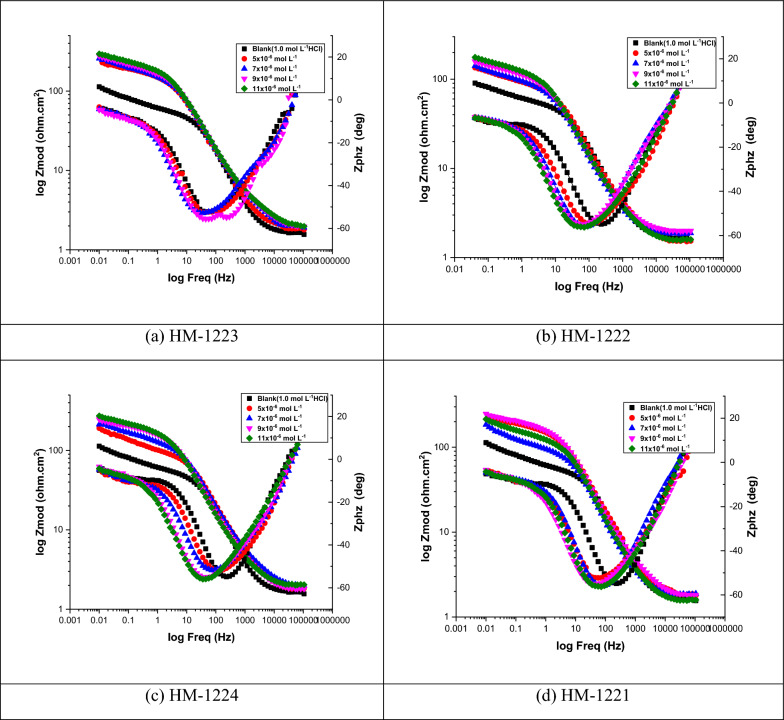
Bode plot for corrosion of CS in 1.0 M HCl and in the existence of various doses of organic constitutions **a-d** at 298 K

EIS is used to investigate the kinetics and the surface characteristics of the electrode processes. To better mimic the non-ideal capacitive behavior of the double layer, double layer capacitance (*C*_dl_) is replaced with a constant phase element (CPE) in the circuit, which is made up of solution resistance (*R*_s_) in series with the parallel combination of charge transfer resistance (*R*_ct_) Fig. 10. According to a previous study [74], the impedance of CPE is as follows:

12 $$Z_{{{\text{CPE}}}} = {\xi \mathord{\left/ {\vphantom {\xi {\left( {i\omega } \right)^{{\text{n}}} }}} \right. \kern-0pt} {\left( {i\omega } \right)^{{\text{n}}} }}$$

where *i* denotes the complex number, *ω* the angular frequency, *ξ* the proportionality factor and n the exponent of the CPE. Nyquist and Bode graphs for the corrosive dissolution of CS in HCl solution with and without varying doses of inhibitors as depicted in Figs. 11, 12, respectively. The Nyquist graphs demonstrated that with an increase in inhibitor dose, the semicircular capacitance diameter is expanded due to the charge transfer phenomena in the solution [75]. EIS variables including charge transfer resistance (*R*_ct_), capacitance of the double layer (*C*_dl_) and *η* (Table 8) showing that the *C*_dl_ values decrease with increasing inhibitor dose, this is due to the adsorption of inhibitors on CS surface leading to formation of a film from the acidic solution [76]. It is clear that *R*_ct_ values rise as the concentration of the inhibiters increase, this due to the increase in the thickness of the double layer as a result of an expansion of the double layer's thickness [77] led to a decrease in dielectric constant [78] and this indicates that ${\eta }_{EIS} \%$ increase. The value of *C*_dl_ can be determined from Eq. 13 [79]:

13 $$C_{{{\text{dl}}}} = Y_{0} \left( {\omega_{{{\text{max}}}} } \right)^{{{\text{n}} - {1}}} = Y_{0} \left( {{2}\pi f_{{{\text{Zim}} - {\text{max}}}} } \right)^{{{\text{n}} - {1}}}$$

where ωmax related to the frequency at which the imaginary impedance in the Nyquist plot is maximum; Y_o_ is CPE and n is CPE exponent. Based on n, CPE can represent (n = 0, Y_o_ = R), capacitor (n = 1, Y_o_ = C), inductance (n = − 1, Y_o_ = L) or Warburg impedance (n = 0, *Y*_o_ = W).

**Table 8:** Parameters gained from EIS in 1.0 M HCl and with the addition of doses of the investigated additives

Inhibitor	Conc., M	*Y*_**o**_**, **µΩ^−1^ s^*n*^ cm^−2^	*n*	*R*_ct_, Ω cm^2^	*C*_dl_, μF cm^−2^	*θ*	*η*_EIS_	Goodness of fit (χ^2^)
**HM-1223**	1.0 mol Lˉ^1^	996	0.999	16 ± 0.1925	992 ± 0.1925	–	–	18.77 × 10^–3^
	5 × 10ˉ^6^	787	0.986	59 ± 0.1234	753 ± 0.1215	0.729	72.9	20.89 × 10^–3^
	7 × 10ˉ^6^	749	0.983	65 ± 0.1324	710 ± 0.1214	0.754	75.4	22.21 × 10^–3^
	9 × 10ˉ^6^	733	0.979	129 ± 0.1147	696 ± 0.1754	0.876	87.6	17.65 × 10^–3^
	11 × 10ˉ^6^	728	0.954	224 ± 0.1754	667 ± 0.1457	0.929	92.9	19.08 × 10^–3^
**HM-1222**	5 × 10ˉ^6^	814	0.969	57 ± 0.1441	737 ± 0.1425	0.719	71.9	20.76 × 10^–3^
	7 × 10ˉ^6^	794	0.962	63 ± 0.1784	705 ± 0.1112	0.746	74.6	19.29 × 10^–3^
	9 × 10ˉ^6^	772	0.959	90 ± 0.1324	688 ± 0.1012	0.822	82.2	19.98 × 10^–3^
	11 × 10ˉ^6^	751	0.930	160 ± 0.1245	640 ± 0.1120	0.900	90.0	16.76 × 10^–3^
**HM-1224**	5 × 10ˉ^6^	824	0.962	53 ± 0.1754	728 ± 0.1245	0.698	69.8	18.54 × 10^–3^
	7 × 10ˉ^6^	803	0.955	62 ± 0.1477	697 ± 0.1325	0.742	74.2	20.88 × 10^–3^
	9 × 10ˉ^6^	798	0.945	70 ± 0.1245	674 ± 0.1124	0.771	77.1	15.56 × 10^–3^
	11 × 10ˉ^6^	762	0.917	156 ± 0.1758	628 ± 0.1425	0.897	89.7	14.77 × 10^–3^
**HM-1221**	5 × 10ˉ^6^	833	0.943	50 ± 0.1985	687 ± 0.1654	0.680	68.0	17.6110^–3^
	7 × 10ˉ^6^	819	0.931	60 ± 0.1875	655 ± 0.1574	0.733	73.3	18.54 × 10^–3^
	9 × 10ˉ^6^	804	0.925	67 ± 0.1625	634 ± 0.1455	0.761	76.1	17.87 × 10^–3^
	11 × 10ˉ^6^	770	0.889	149 ± 0.1524	587 ± 0.1554	0.893	89.3	21.55 × 10^–3^

Equation 14 is utilized to calculate the inhibition efficiency based on the polarization resistance [80, 81]:

14 $${\eta }_{EIS}=\frac{{R}_{ct (inh)} - {R}_{ct}}{{R}_{ct (inh)}}\times 100$$

where *R*_ct_ and *R*_ct(inh)_ refer to the charge transfer resistance without and with the addition of inhibitors, respectively. The results from EIS are compatible with those acquired from the PDP analysis. The standard evaluation criteria for determining which of these compounds agreed the best with the data used: low chi-square errors (χ^2^ about 10^–4^) and low 5% for allowable elemental errors in fitting mode. Therefore, in this case, the circuit in use is acceptable. The ${\eta }_{\text{EIS}}\text{ \% of these compounds follows the following order}:$ **HM-1223** > **HM-1222** > **HM-1224** > **HM-1221**.

### Surface analysis study

#### Scanning *electron* microscope (SEM) analysis

**Fig. 13:**
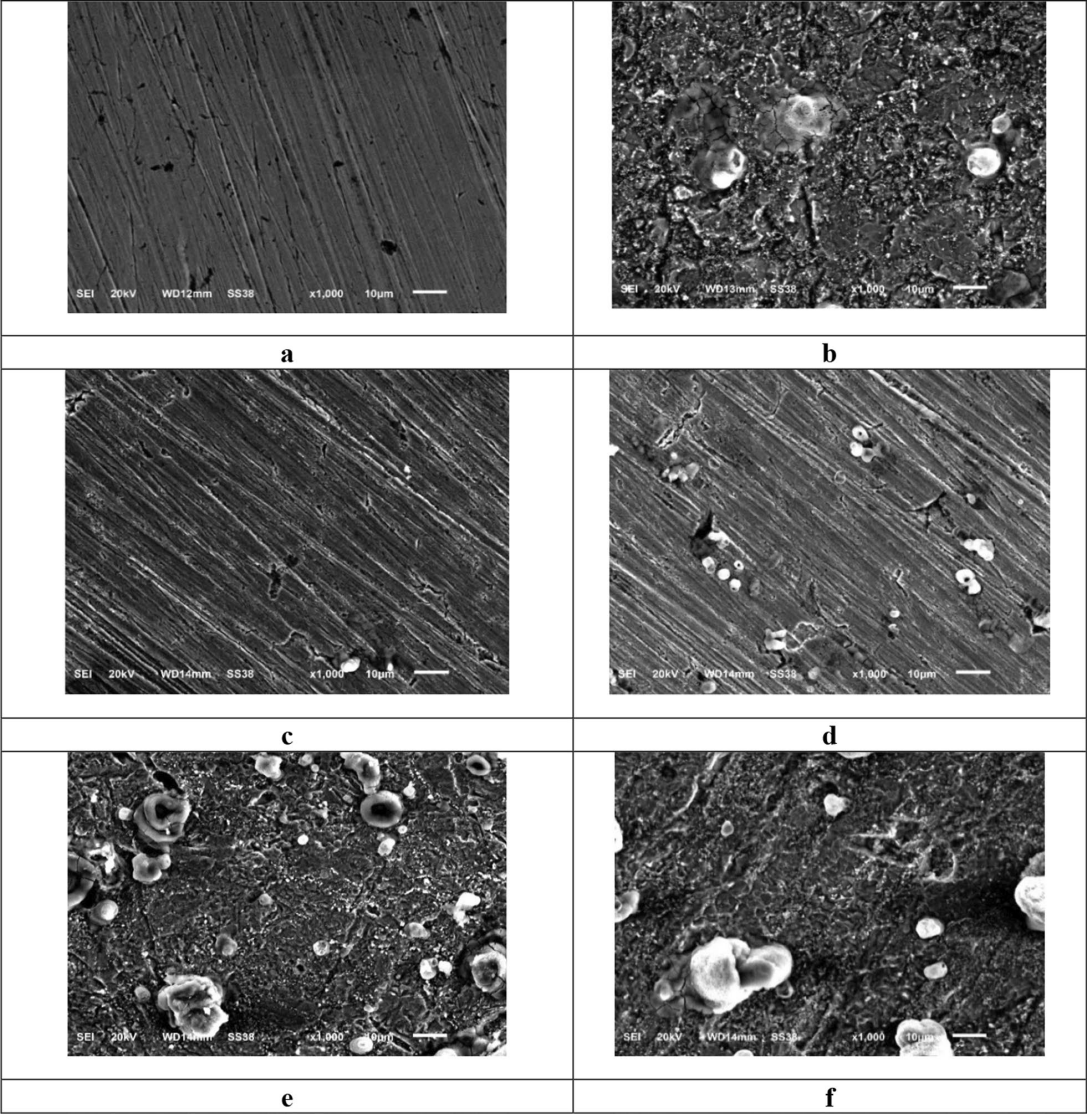
SEM images for CS smooth surface (**a**), then after 24 h immersion in 1.0 M HCl (**b**) and in the existence of 11 × 10^−6^ M of inhibitors (**c-f**)

The morphology of the CS surface was evaluated using SEM to determine whether the inhibition was caused by the growth of an organic coating. The SEM images for CS surface immersed HCl and with inhibited solutions are illustrated in Fig. 13a–f. The CS sample's surface was smoother before immersion (Fig. 13a), but due to the acidic solution's powerful attack **(**Fig. 13b), the surface became very coarse with significant corrosion and cracks distributed throughout after immersion in HCl **(**Fig. 13b). But in the presence of organic inhibitors, which have a softer and smoother surface (Fig. 13c, f), the damage has been reduced. The development of a protective organic suppressive coating on the metal's surface is indicated by this smoother surface morphology [82, 83].

#### EDX studies

**Fig. 14:**
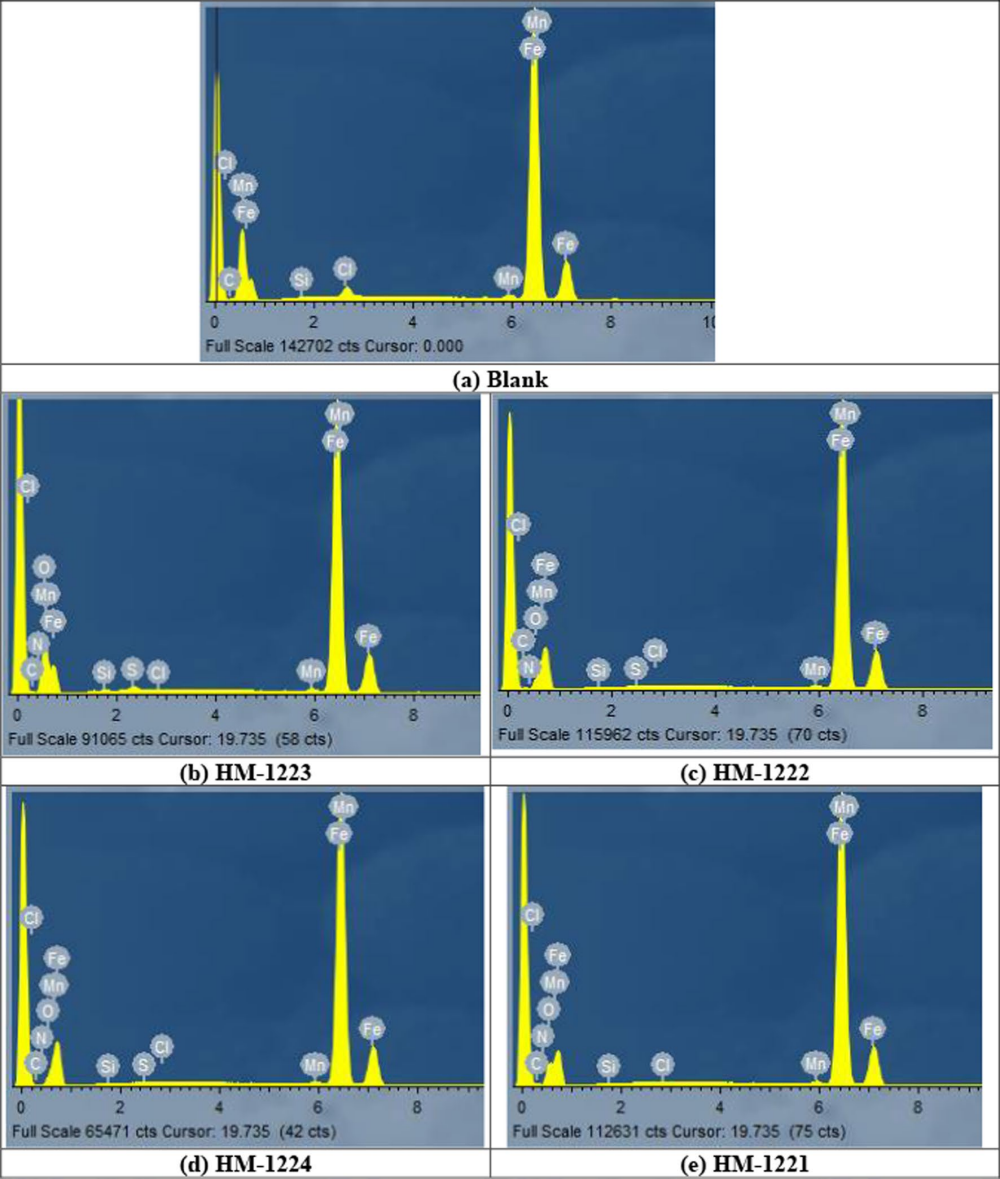
EDX spectra of CS (**a**) after 24 h immersion in 1.0 M HCl (**b**) and in the existence of 11 × 10^−6^ M of inhibitors (**b**–**e**)

Figure 14 depicts the EDX spectra that demonstrate the specific peaks of certain elements constituting the CS afterward 24 h in the unprotected and protected 1.0 M HCl. EDX spectra in the existence of the maximum dose of the chemicals display extra lines of carbon, nitrogen, sulfur and oxygen owing to the layer of the adsorbed chemicals on CS. From Table 9**,** it was found that [84]:

- 1-Intensities of C, O, S and N signal are enhanced and this due to N, C, S and O atoms present in the chemical composition of the inhibitors, indicating adsorption of the chemicals molecules on the surface of CS.
- 2-Fe peaks are suppressed in the existence of the inhibitors which is because of overlying inhibitor film [85].

**Table 9:** Surface characteristics (wt. %) of CS both earlier and later dispersion in 1.0 M HCl with and without of 11 × 10^−6^ M of inhibitors

(Mass %)	Fe	C	Mn	Cl	O	S	N
Blank	82.29	3.97	0.81	0.63	12.3	–	–
**HM-1223**	53.11	6.30	0.72	0.11	23.21	15.26	1.29
**HM-1222**	54.23	5.20	0.66	0.10	23.72	14.92	1.17
**HM-1224**	61.04	9.76	0.36	0.11	27.72	–	1.01
**HM-1221**	61.99	9.87	0.31	0.13	26.72	–	0.98

#### AFM analysis

**Fig. 15:**
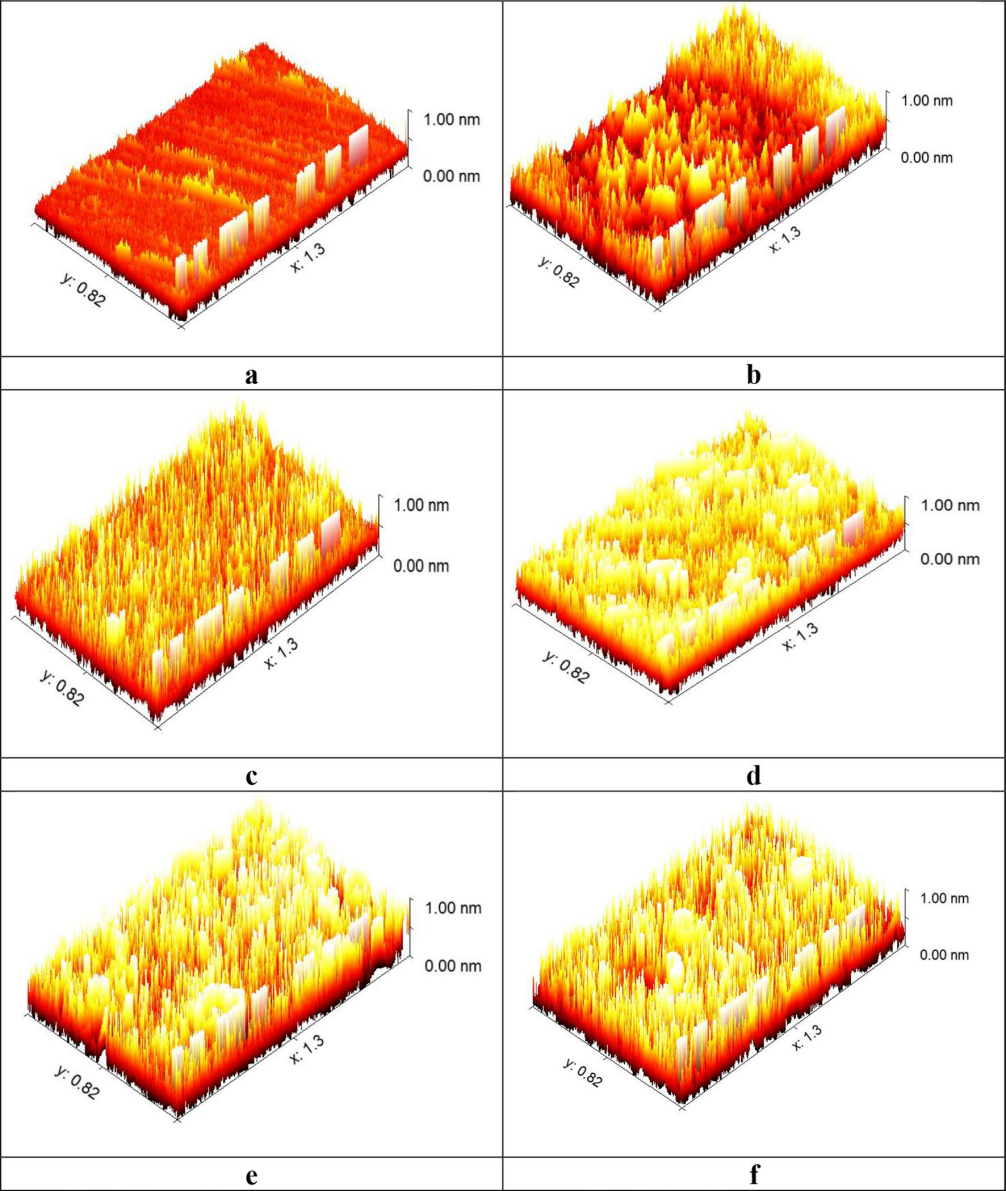
**a** Represent smoother image CS surface taken by AFM, whereas, image **b** indicates what happened after immersion in HCl only, while, images from (**c** to **f**) refer to the presence of 11 × 10^−6^ M of inhibitors

AFM is an effective method for examining topography of the surface which confirms the adsorption of inhibitors on the surface of the corroding metal. Figure 15a–f displays three-dimensional AFM images of the CS surface before and after the immersion of inhibitors. The roughness of the CS surface related to uninhibited solution in HCl only is 879.3 mm as average (Fig. 15b), and the surface with polishing roughness is 22.3 mm (Fig. 15a). Nevertheless, in the existence of inhibited scaffolds (Fig. 15c–f) at the highest chosen dose (11 × 10^−6^ M), the average roughness declines to 101.3 mm for (**HM-1223**), 133.2 mm for (**HM-1222**), 187.1 mm for (**HM-1224**) and 196.7 mm for (**HM-1221**). These evidences show that the CS surface is smoother in the presence of inhibitors compared the absence of inhibitors due to the establishing a defensive coating adsorbed from the molecules of inhibitors that protects CS surface [86].

**Fig. 16:**
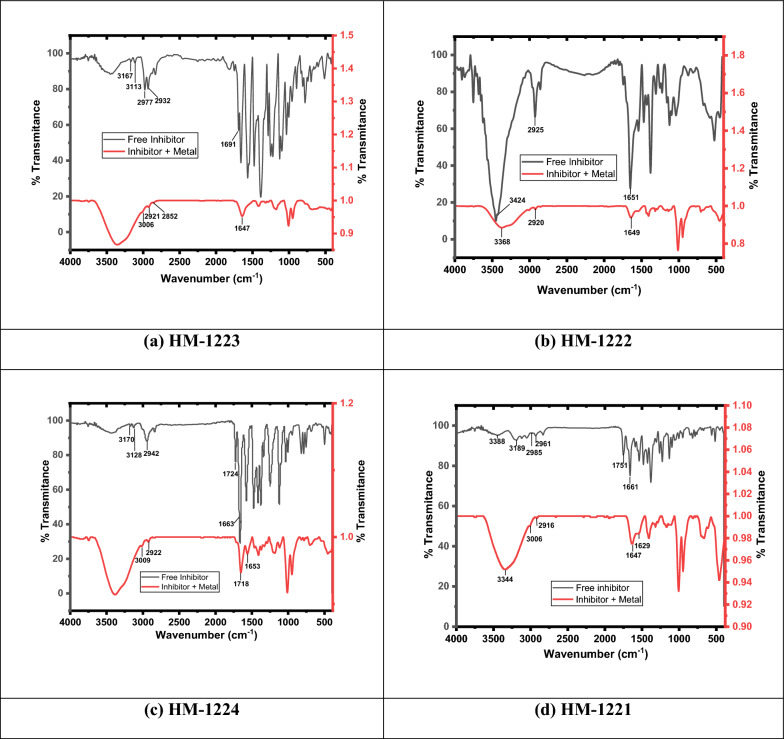
FT-IR spectra of **a** **HM-1223**, **b** **HM-1222**, **c** **HM-1224**, and **d HM-1221**

#### FTIR technique

FT-IR is a crucial analytical tool to understanding efficacious groups and characterizing bonding with metal**.** Certain peaks of the IR spectra are corresponding to the function groups of the substances under investigation. The characteristic peaks of active function groups for free organic compounds before (pure inhibitors) and the other peaks in the presence of these compounds after immersing CS for 24 h in 1.0 M HCl + 11 × 10^−6^ M at 298 K were attained and compared to each other (Fig. 16). The data of FT-IR showed that: the peaks of the function groups of the adsorbed chemicals show a tightly shifting, this confirmed the complex formation between Fe metal and inhibitors [68] and consequently, these substances have the potential to operate as corrosion inhibitors [87, 88]**.**

### Quantum chemical calculations

**Fig. 17:**
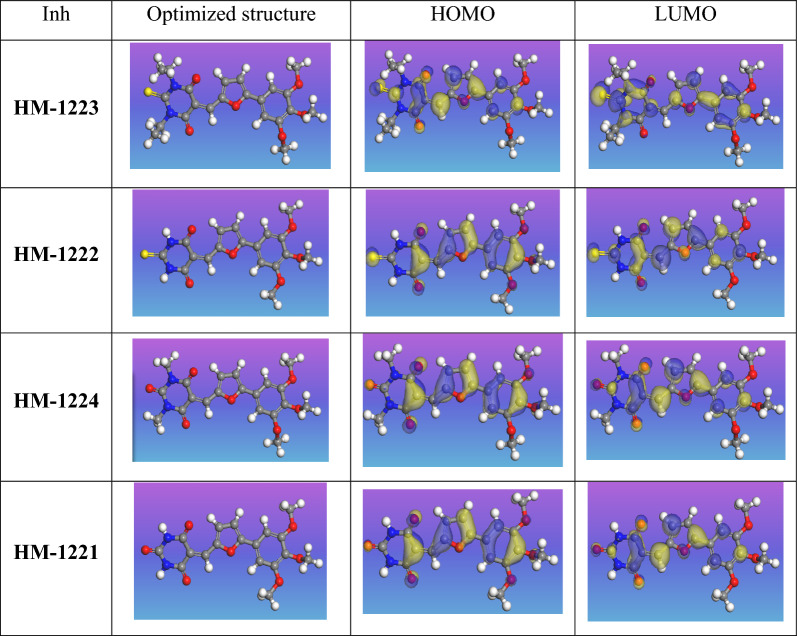
HOMO and LUMO electron density maps for the studied inhibitors

To anticipate the configuration and electron dispersion of trimethoxyphenylfurylidene-pyrimidinone derivatives, quantum chemical computations are employed. The evaluation of molecular reactivity is commonly performed using density functional theory (DFT). Figure 17 shows the optimized structures of the inhibitors studied. Whereby, *E*_HOMO_ and *E*_LUMO_ (FMOs) are crucial descriptors in chemistry for studying the chemical reactivity in various reactions, the donor–acceptor interaction between adsorbed molecules and FMOs of adsorbent atoms can give valuable insights in exploring most chemical interactions, particularly those involving compound adsorption such as corrosion inhibition properties. An increase in E_HOMO_ values often indicates a molecule's greater ability to donate electrons to an acceptor molecule with vacant molecular orbitals. Conversely, a lower *E*_LUMO_ value often associates with a higher capacity accept electrons by the reacting species. As a result, a lower *E*_LUMO_ is anticipated that a molecule has a greater tendency to gain electrons in specific interactions. In this sense, *E*_HOMO_ can measure ionization potential and a species' tendency to undergo electrophilic attack, while *E*_LUMO_ is indicative of its susceptibility to nucleophilic attack. Therefore, an increase in E_HOMO_ and decrease in *E*_LUMO_ are expected to be typical of high corrosion inhibition properties of compounds by promoting their adsorption on metallic surfaces through chemisorbed film formation. The difference between *E*_LUMO_ and *E*_HOMO_ (Δ*E*) is a crucial stability index that is associated with corrosion inhibition capabilities in corrosive and tribological systems [89, 90].

A small energy gap between HOMO and LUMO orbitals suggests a soft nature, while a large gap indicates a hard nature. Whereby, *η* values is enhanced this is commitment to increase the value of *E*_HOMO_ and reduction in both *E*_LUMO_ and Δ*E*. Table 10 lists the results of quantum calculations, such as both E_HOMO_, E_LUMO_ and energy gap (Δ*E*), while other quantum chemical parameters [90, 91]. Based on the values on Table 10, the trend in the quantum chemical parameters shows that the increasing order of inhibition follows: **HM-1223** > **HM-1222** > **HM-1224** > **HM-1221**.

**Table 10:** List of quantum chemical parameters on the investigated inhibitor compounds

Inhibitor	HM-1223	HM-1222	HM-1224	HM-1221
*E*_HOMO_ (ev)	− 3.765	− 5.477	− 5.366	− 5.425
*E*_LUMO_ (ev)	− 2.342	− 3.809	− 3.662	− 3.680
Δ*E* = *E*_LUMO_- *E*_HOMO_	1.423	1.668	1.705	1.745
*ƞ* = Δ*E* /2	0.711	0.834	0.852	0.872
*σ*(S) = 1/ ƞ	1.406	1.199	1.173	1.146
*π* = (*E*_HOMO_ + *E*_LUMO_)/2	− 3.053	− 4.643	− 4.514	− 4.552
*X* = − *π*	3.053	4.643	4.514	4.552
∆*N* max	2.146	2.783	2.648	2.609
∆*N* (FET)	1.242	0.106	0.180	0.153
*ω*	6.553	12.922	11.952	11.877
*ε*	0.153	0.077	0.084	0.084
Δ*E* Back-donation	− 0.178	− 0.209	− 0.213	− 0.218

The effect of corrosion inhibition effects of the four inhibitors were found to be consistent with the decreasing order of energy gap and E. In contrast to **HM-1222** molecule, which has two N–H hydrophilic groups, **HM-1223** compound, which has a furan ring and two ethyl groups, has stronger electron donating capacity and lipophilic qualities. Additionally, introducing (S) atom enhances capacity of molecules to give electrons by sharing their lone pair. While **HM-1224** and **HM-1221** has lower electron donating ability than **HM-1223** and **HM-1222** due to the weaker impact of their (O) atom in to donate electrons compared to S atom.

### Monte carlo simulation studies

**Fig. 18:**
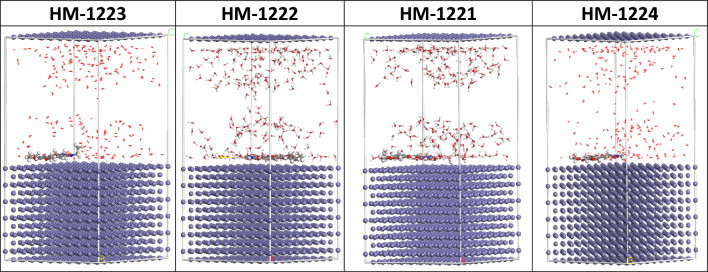
The most suitable adsorption configuration of four inhibitors on Fe (1 1 0) using adsorption locator module

MC simulation was used to visualize the interaction between the four inhibitor molecules with the CS surface and the adsorption mechanism. Figure 18 shows the most possible adsorption configurations of pyrimidinone molecules on the CS. This could be achieved via the adsorption locator module, which exhibits smooth disposition and provides an improvement in adsorption with the greatest surface coverage. The data that were ascertained via MC simulations are listed in Table 11. The unrelaxed and relaxed adsorption energies of four inhibitors were summarized in Table 11 before and after the geometry optimization procedure. It is found that **HM-1223** has a higher negative value of adsorption energy equal to (− 3.288385e + 003 kcal mol^−1^), followed by **HM-1222** (− 3.205497e + 003 kcal mol^−1^), then **HM-1224** (− 3.199657e + 003), while **HM-1221** has the lowest value equals to (-3.130904e + 003 kcal mol^−1^). Furthermore, **HM-1223** has implying robust adsorption on the CS surface and form a fixed adsorbed film. The dE_ads_/dNi values illustrate the metal-adsorbate configuration’s energy if one of the adsorbates is eliminated. **HM-1223** inhibitor has superior adsorption than other inhibitors, as evidenced by the fact that its dE_ads_/dNi value is higher (-259.44618281 kcal mol^−1^) than **HM-1222**, **HM-1224**, and **HM-1221**. Furthermore, the dE_ads_/dNi value for water is low when compared to the studied inhibitors values, indicating that the studied inhibitors were adsorbed more strongly than water molecules on the CS surface, supporting the replacement of water molecules with pyrimidinone inhibitors. Furthermore, it can be summarized that these MC results correspond well with the quantum chemical calculations as well as the experimental data [10, 51].

**Table 11:** The calculated data utilizing MC simulations for the adsorption inhibitors on Fe (1 1 0)

Structures	Adsorption energy/Kcal mol^−1^	Rigid adsorption energy/kcal mol^−1^	Deformation energy/kcal mol^−1^	dEads/dNi: Inhibitor mol^−1^	dEads/dNi: Water mol^−1^
Fe (1 1 0) 1223 Water	−3.288385e + 003	− 3.452641e + 003	164.25625140	− 259.44618281	− 7.04265953
Fe (1 1 0) 1222 Water	− 3.205497e + 003	− 3.368687e + 003	163.19066031	− 248.85991961	− 7.92810123
Fe (1 1 0) 1224 Water	− 3.199657e + 003	− 3.362333e + 003	162.67538061	− 236.86523223	− 7.92725663
Fe (1 1 0) 1221 Water	− 3.130904e + 003	− 3.293055e + 003	162.15187138	− 148.83457928	− 8.85918095

### Mechanism of inhibition

**Fig. 19:**
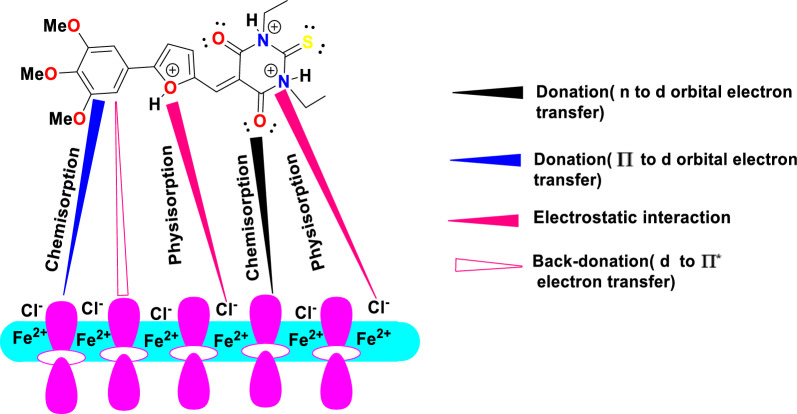
Mechanism of inhibition of compound **(HM-1223**, **5c**)

The adsorption process is influenced by the inhibitors’ chemical composition, surface charge, and internal charge distribution. Generally, chemisorption and physisorption-two different ways whereby inhibitor compounds can adsorb on the surface of CS are considered. Organic molecules can be adsorbed through physisorption. The electronegative donor atoms N, O, S, and π-electrons of the aromatic ring in the compounds under investigation effectively facilitate the adsorption of inhibitors onto the surface of CS. Consequently, by hydration chloride ions adsorbed on the metal surface which led to allocate the negative charges, on the other hands, acidic medium acts as positively hydrogen donating atoms. Besides, electrostatic interaction (physisorption) was occurred between positively protonated organic molecules and negatively chloride anions adsorbed on the surface of CS [92]. This surface adsorption results in a protective coating that repels water from the metal’s surface and shields it from corrosion. The development of organic derivatives' adsorption was confirmed by AFM and SEM results. The inhibitors tested in previous experiments can be ranked in terms of inhibition efficiency as **HM-1223** > **HM-1222** > **HM-1224** > **HM-1221**. Due to the two ethyl groups in **HM-1223**, which enhance the molecular size of the compound and act as atom donors, it is thought that **HM-1223** is more complex than **HM-1222**. Due to its higher molecular size, **HM-1224** is superior to **HM-1221** (Fig. 19).

## Conclusion

The newly synthesized derivatives act as efficient inhibitors for CS in 1.0 M HCl between 303 and 323 K. The η improved with an increase in derivative concentrations and decreased with an increase in temperature by 5.0% with increasing temperature. The polarization curves indicate that the investigated inhibitors acted as mixed-type inhibitors, impacting both anodic and cathodic processes without changing the corrosion mechanism. These molecules are adsorbed spontaneously on CS surface based on impedance tests and according to Langmuir adsorption isotherm. The adsorption of these derivatives is of mixed type (Physical and Chemical) but mainly physical. The presented theoretical result is in full agreement with the experimental ones. *η* % of these investigated compounds are in the following order: **HM-1223 **> **HM-1222** > **HM-1224** > **HM-1221**. A corrosion mechanism based on mixed type of these derivatives onto CS surface is proposed.

## Supplementary Information

Supplementary material 1

## Data Availability

All data and analysis during this study are available in this article and its supplementary file.
