# Supplementary material for: Synthesis of new binary trimethoxyphenylfuran pyrimidinones as proficient and sustainable corrosion inhibitors for carbon steel in acidic medium: experimental, surface morphology analysis, and theoretical studies
Source: BMC Chem. 2024 Sep 20;18(1):182. doi: 10.1186/s13065-024-01280-6 (PMC11414101; doi:10.1186/s13065-024-01280-6)
Supplement: Supplementary file 1 — Supplementary material 1 [file 13065_2024_1280_MOESM1_ESM.docx]

**Synthesis of new binary trimethoxyphenylfuran pyrimidinones as proficient and sustainable corrosion inhibitors for carbon steel in acidic medium: Experimental, surface morphology analysis, and theoretical studies**

Hajar A. Ali^a^, Ahmed. A. El-Hossiany^a,b^, Ashraf S. Abousalem^c^, Mohamed A. Ismail^a^, Abd El-Aziz S. Fouda^**,a^, Eslam A. Ghaith^*a^

^a^ Chemistry Department, Faculty of Science Mansoura University, Mansoura, Egypt,

^b^ Delta for Fertilizers and Chemical Industries, Talkha, Egypt.

^c^ Quality Control Laboratory, Operations Department, Jotun, Egypt

*Corresponding author:* ***Eslam A Ghaith*** [abdelghaffar@mans.edu.eg](mailto:abdelghaffar@mans.edu.eg), Tel: +2010244410784

*Cocorresponding author*: ***Abd El-Aziz S. Fouda*** [asfouda@hotmail.com](mailto:asfouda@hotmail.com), Tel: +2 01006450885

**Supplementary Material**

**I. Figures**

**1.1. Figures S1: IR Spectra of the new furylidene-pyrimidines 5a-d.**

**1.2. Figures S2: NMR Spectra of the new furylidene-pyrimidines 5a-d.**

**1.3. Figures S3: Mass Spectra of the new furylidene-pyrimidines 5a-d.**

**1.1. Figures S1: IR Spectra of the new furylidene-pyrimidines 5a-d.**

**
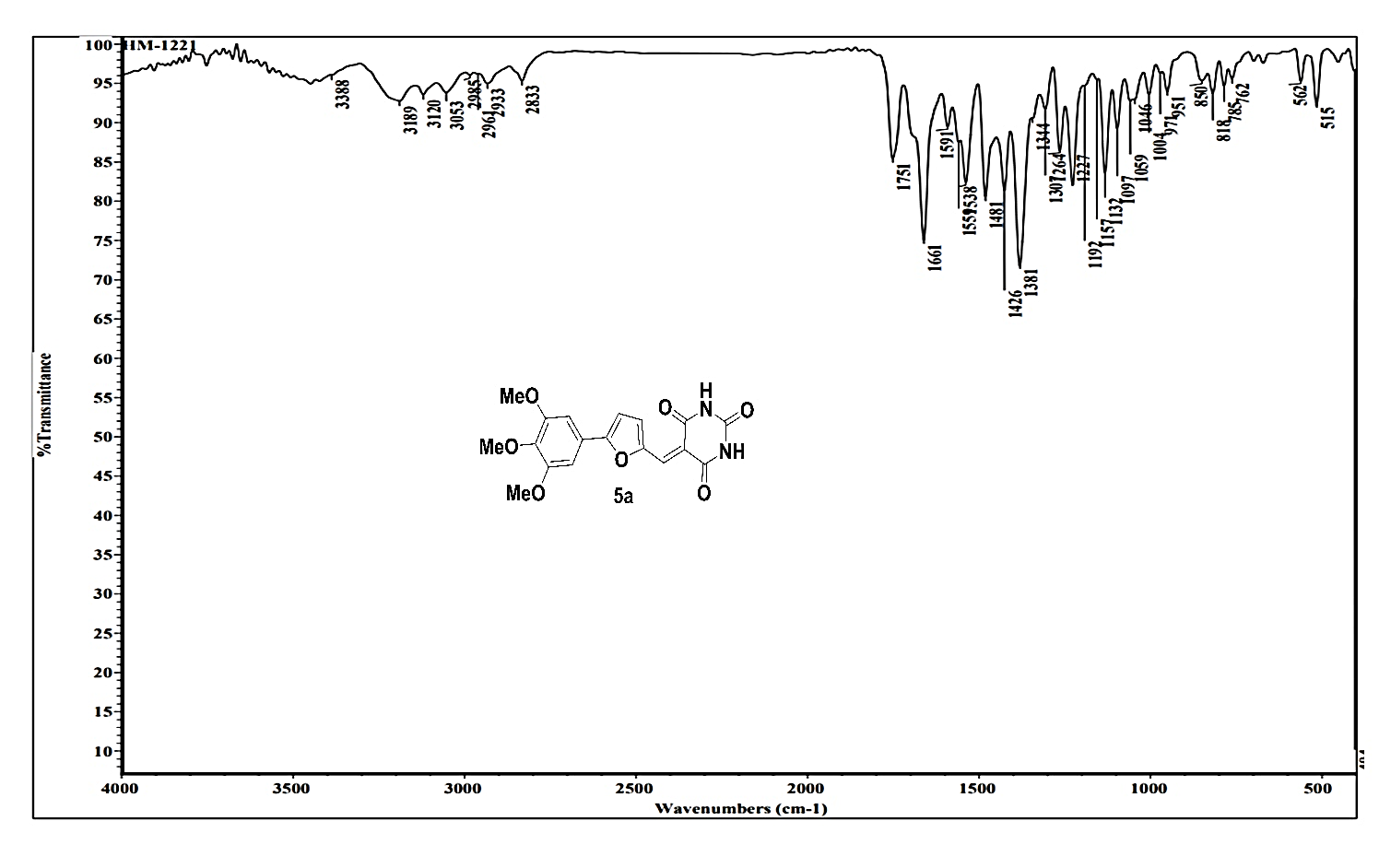
**

**IR Spectrum for compound HM-1221**

**
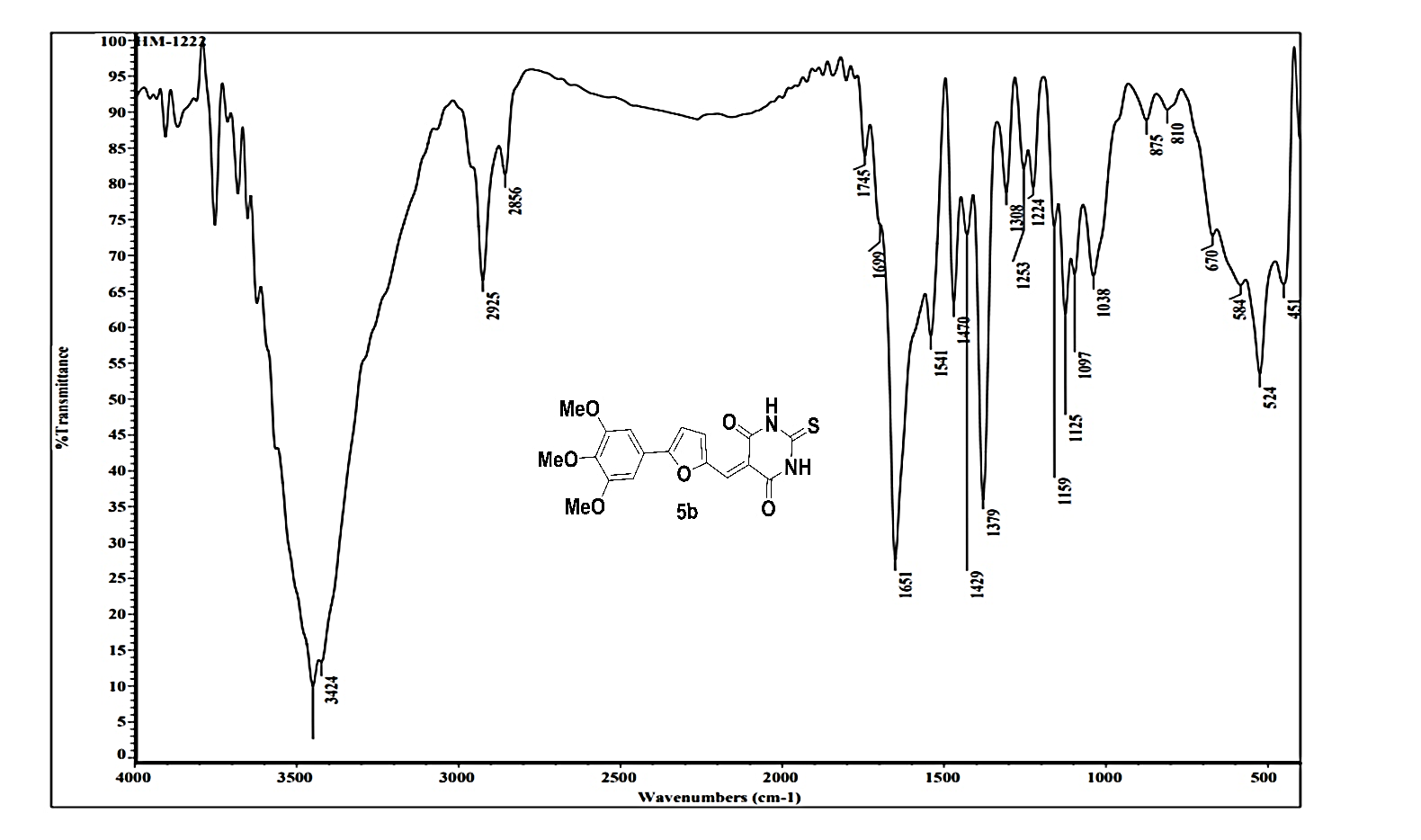
**

**IR Spectrum of compound HM-1222**

**
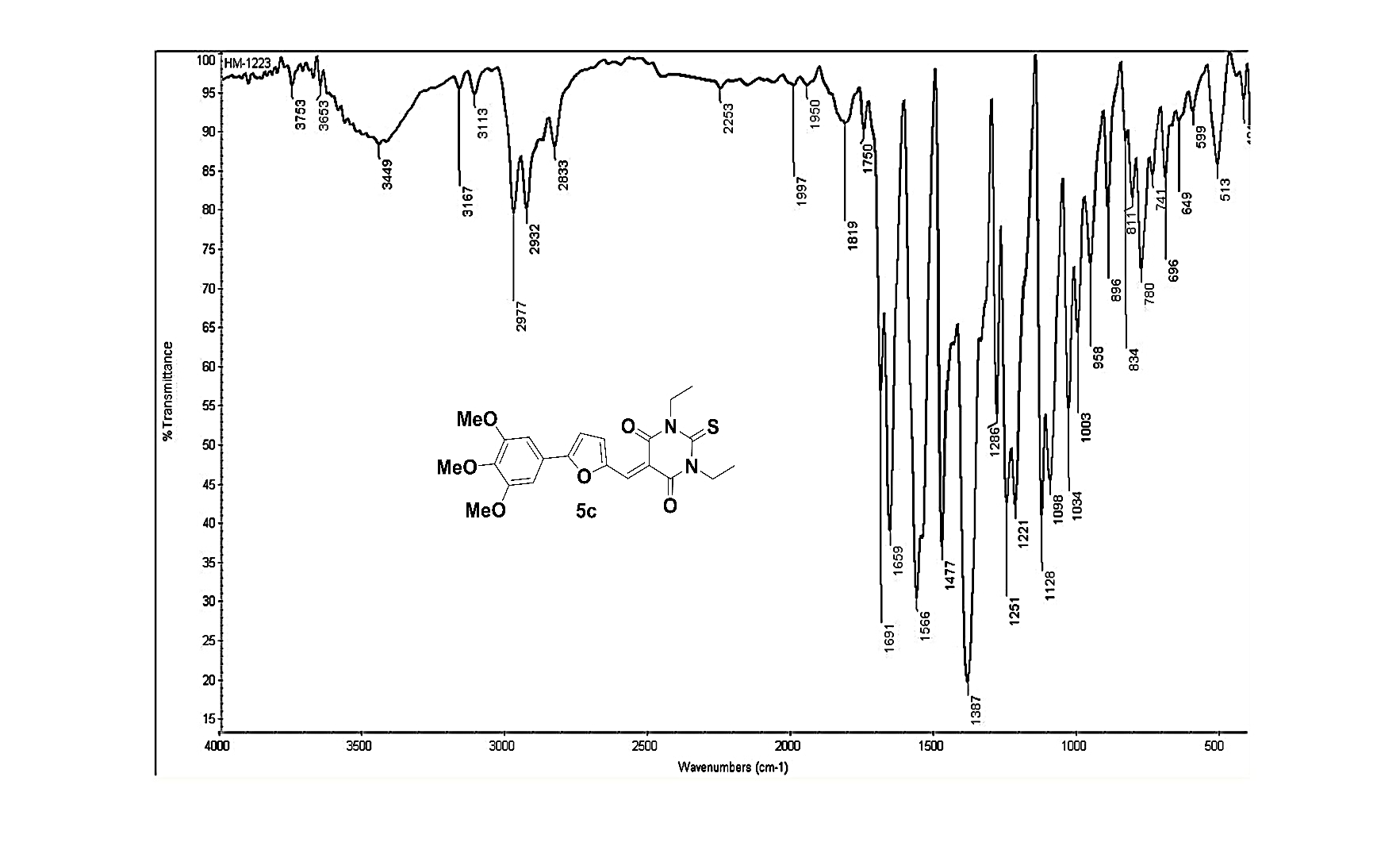
**

**IR Spectrum of Compound HM-1223**

**
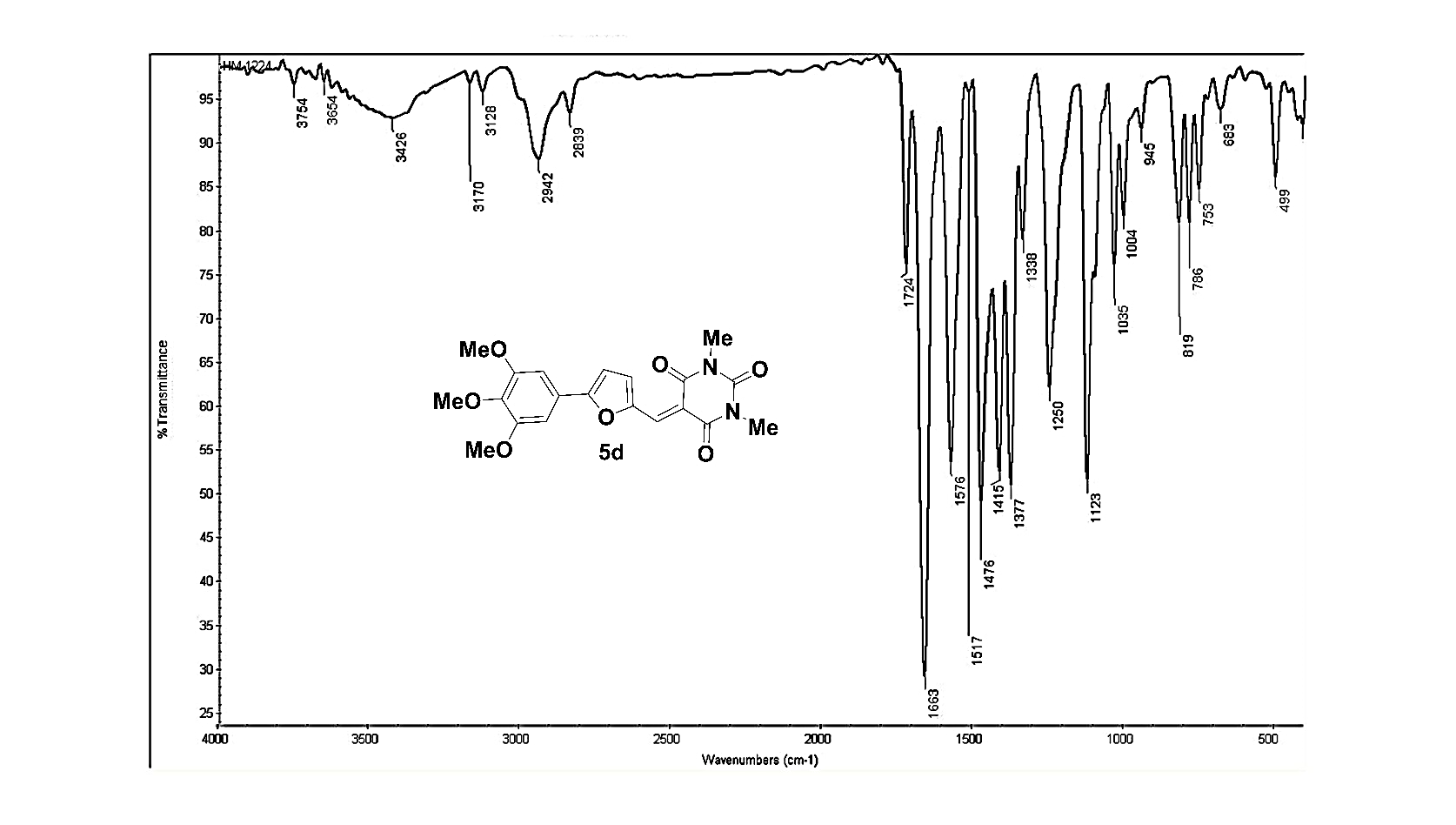
**

**IR Spectrum of compound HM-1224**

**1.2. Figures S2: NMR Spectra of the new furylidene-pyrimidines 5a-d.**

**
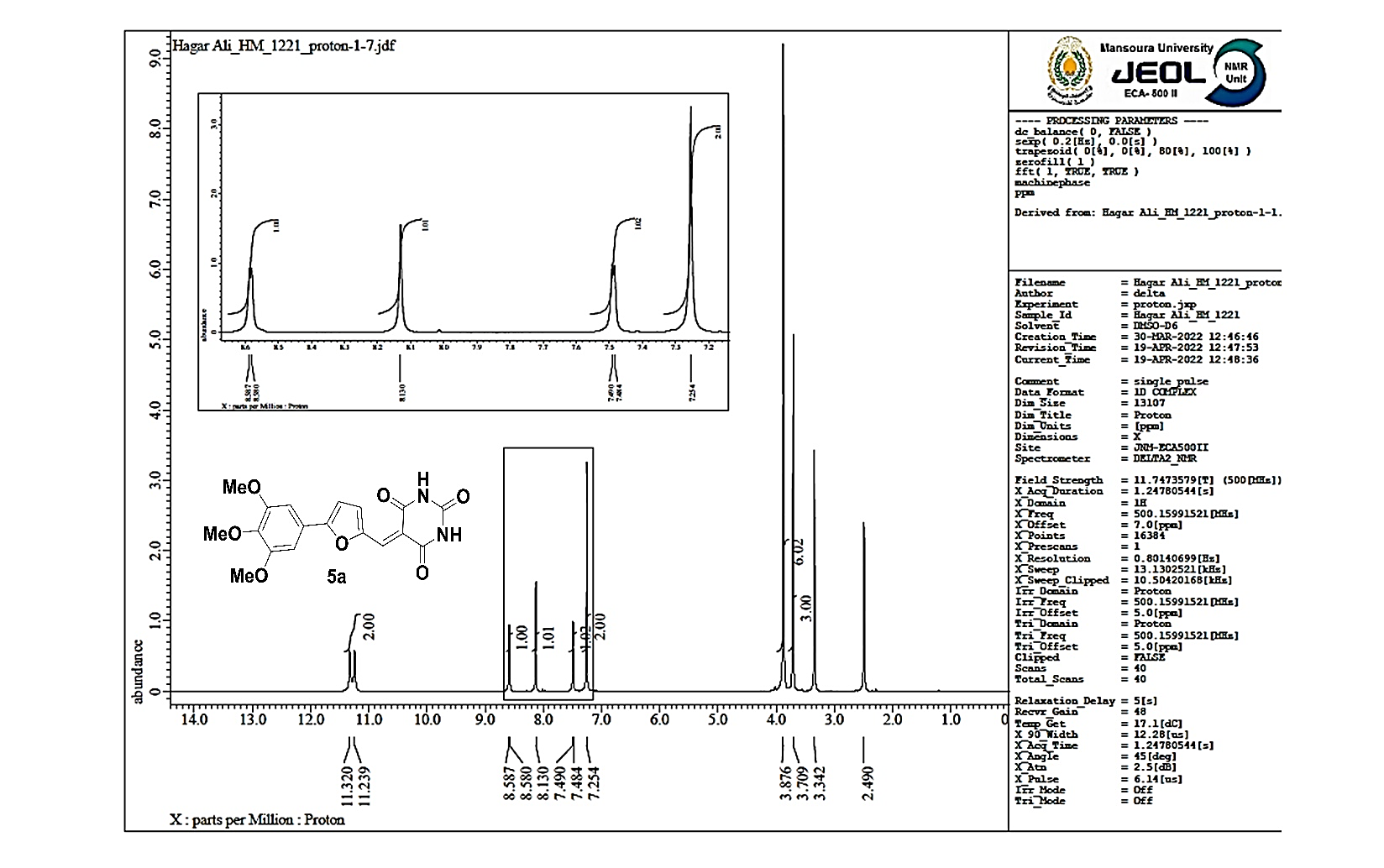
**

**^1^H-NMR (DMSO-*d*_6_)/JEOL 500 *MHz* of compound HM-1221**

**
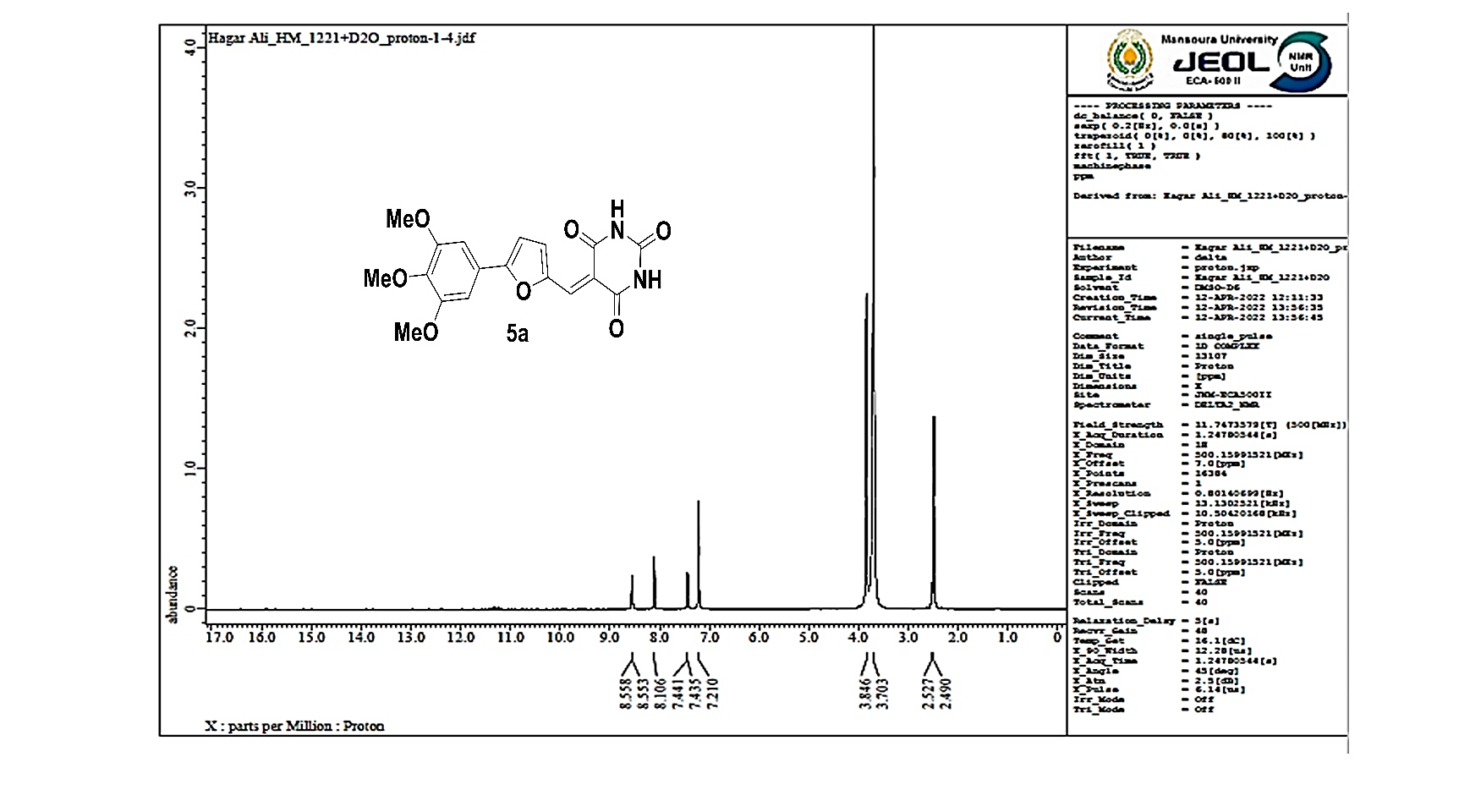
**

**^1^H-NMR (D_2_O/DMSO-*d*_6_)/JEOL 500 *MHz* of compound HM-1221**

**
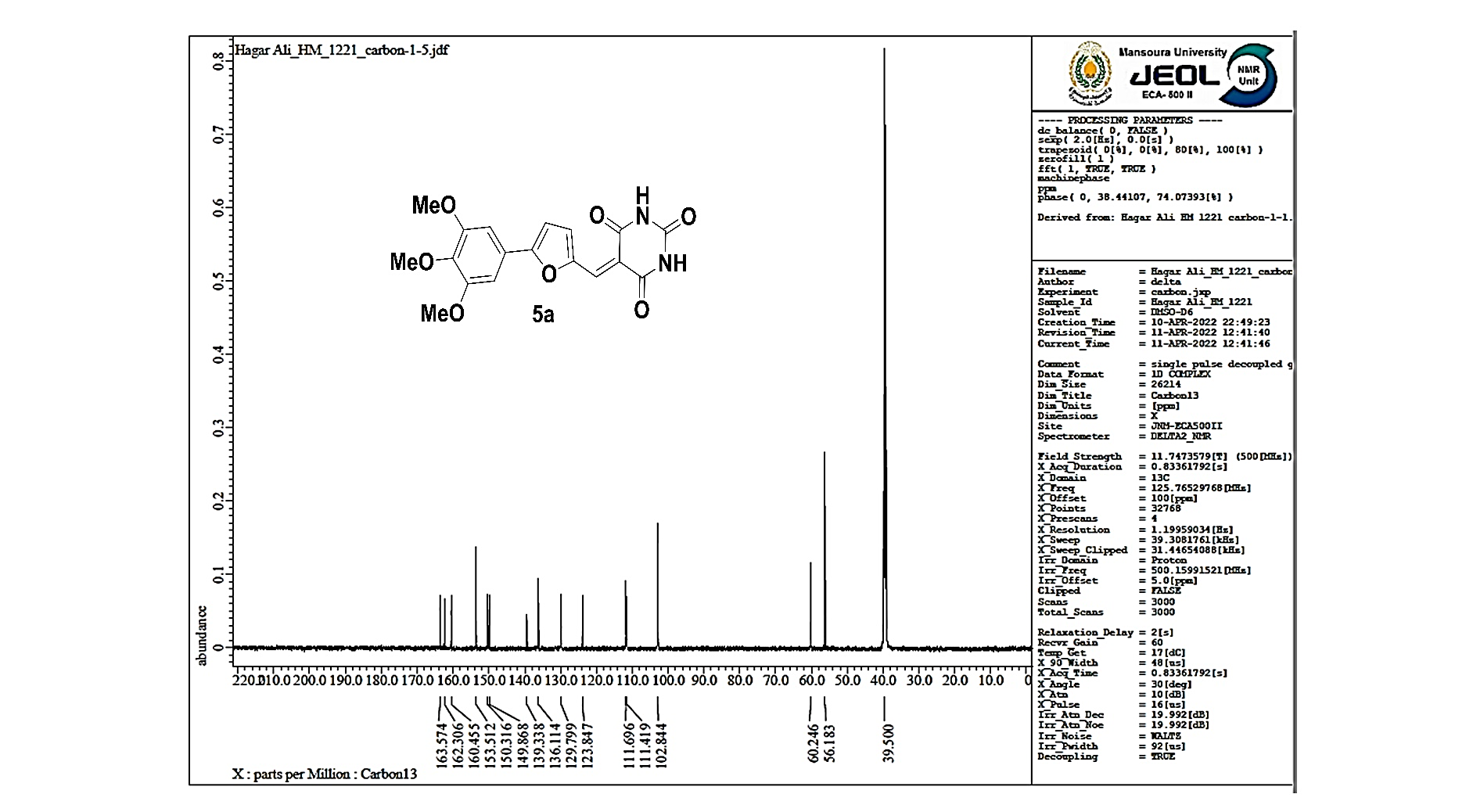
**

**^13^C-NMR (DMSO-*d*_6_)/JEOL 125 *MHz* of compound HM-1221**


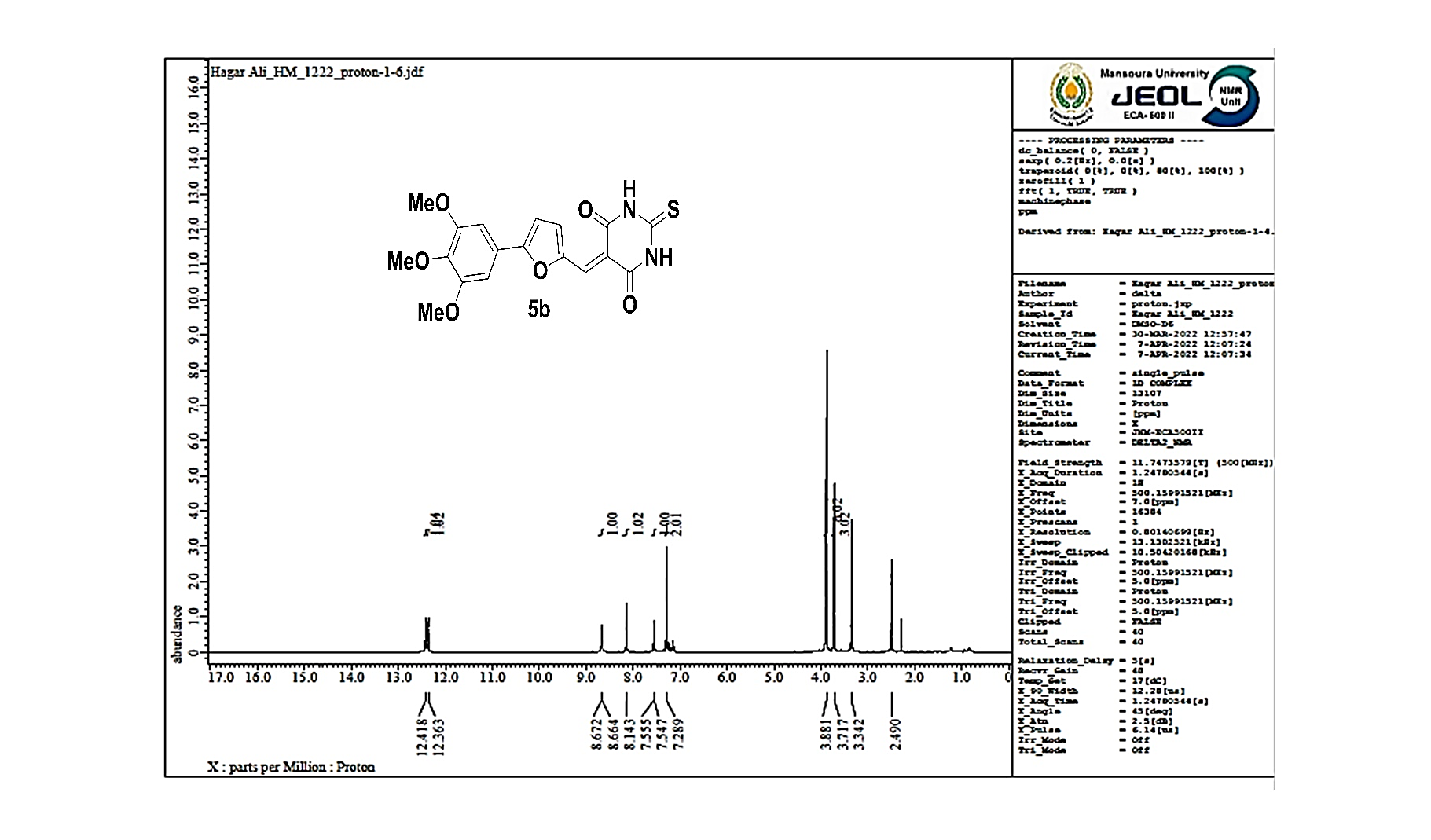


**^1^H-NMR (DMSO-*d*_6_)/JEOL 500 *MHz* of compound HM-1222**

**
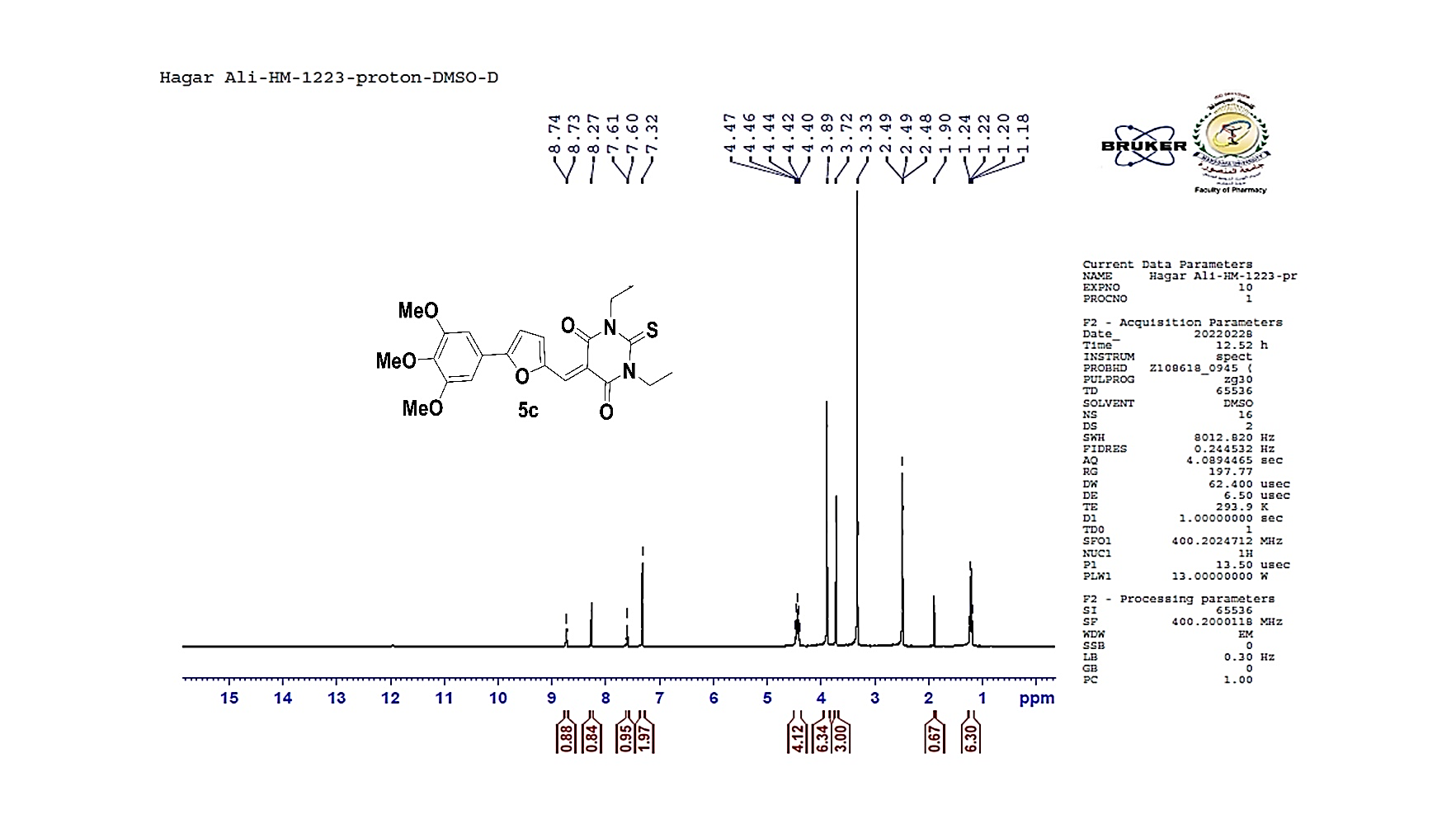
**

**^1^H-NMR (DMSO-*d*_6_)/Bruker 400 *MHz* of Compound HM-1223**


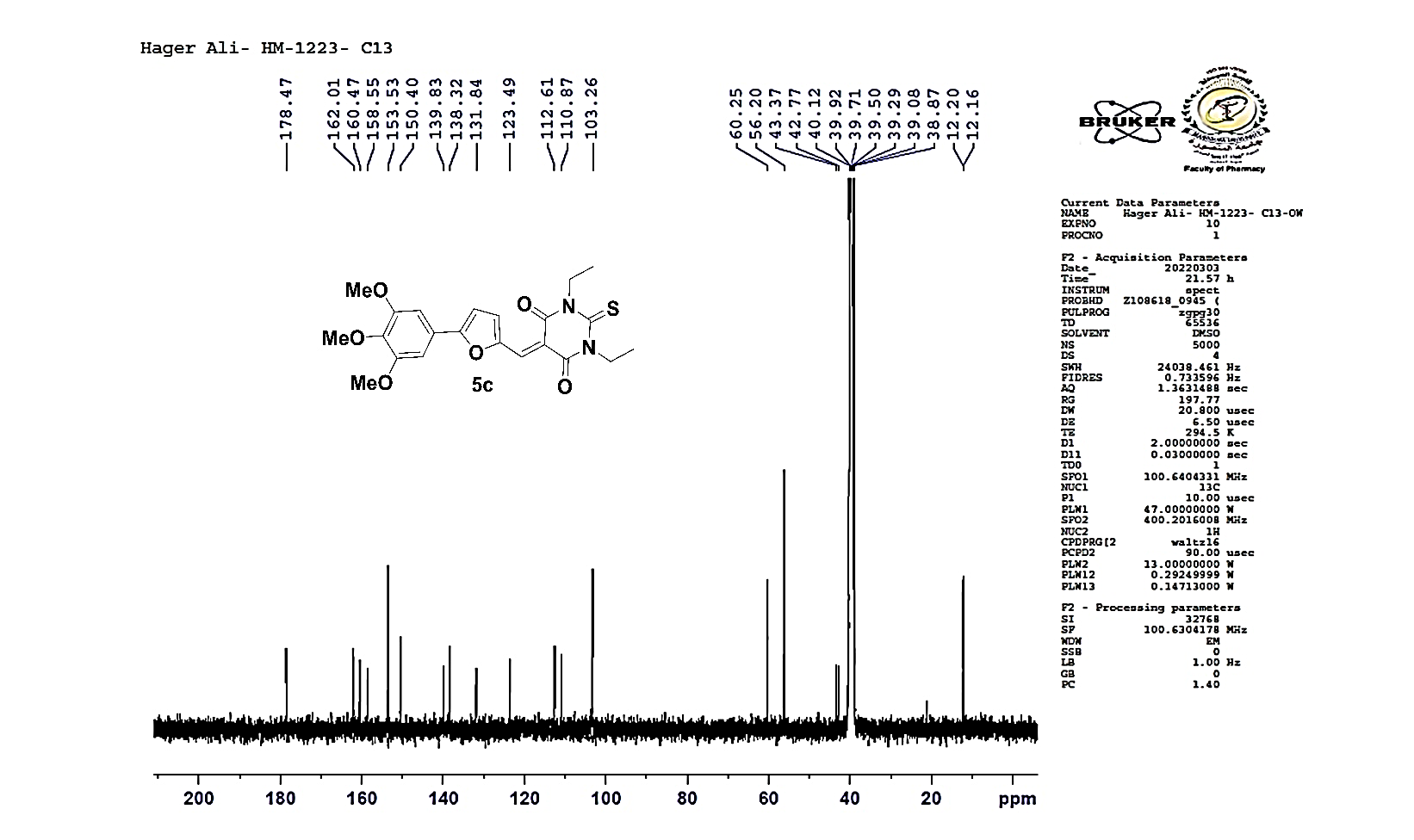


**^13^C-NMR (DMSO-*d*_6_)/ Bruker 100 *MHz* of compound HM-1223**

**
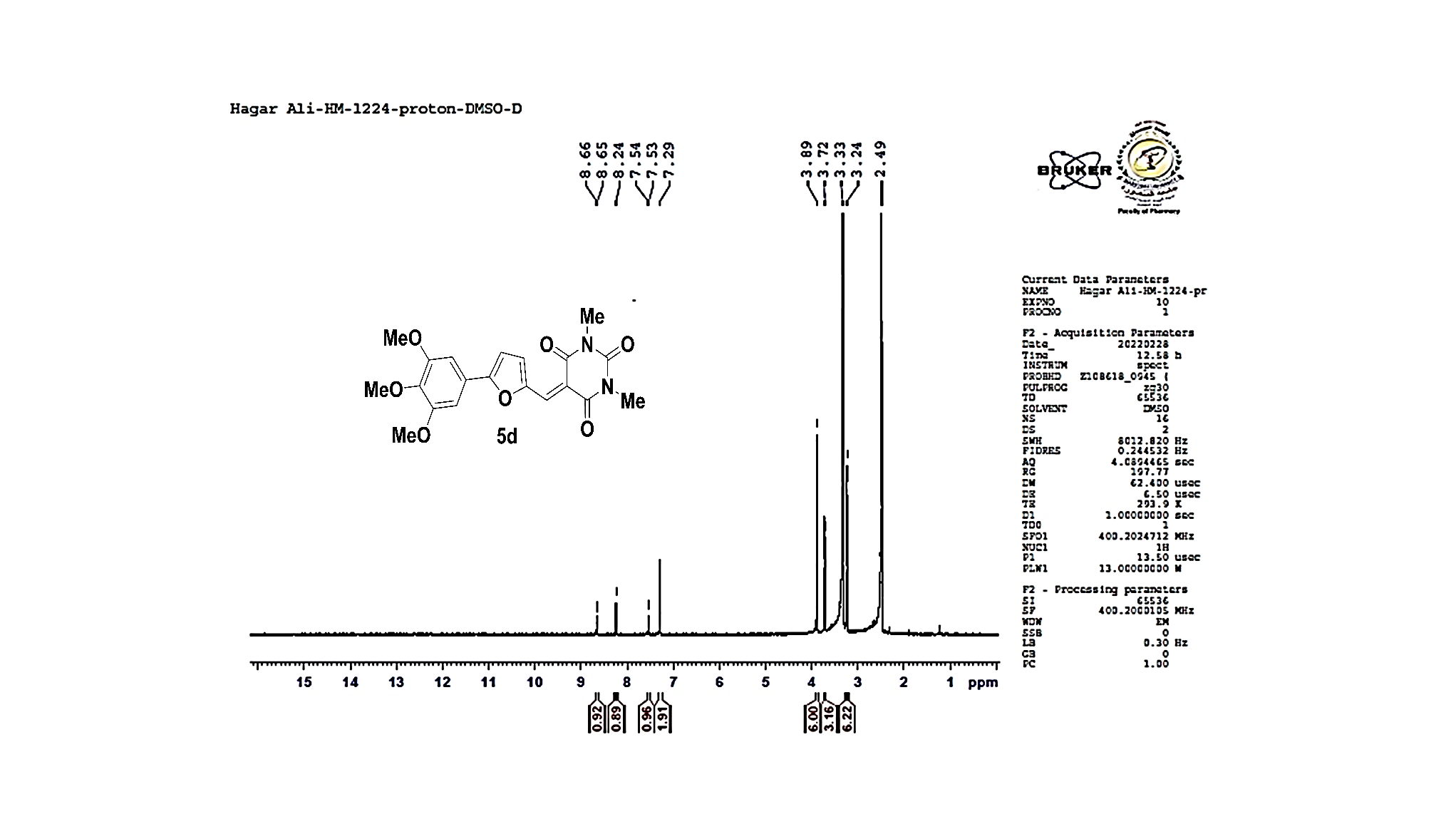
**

**^1^H-NMR (DMSO-*d*_6_)/Bruker 400 *MHz* of compound HM-1224**

**1.3. Figures S3: Mass Spectra of the new furylidene-pyrimidines 5a-d.**

**
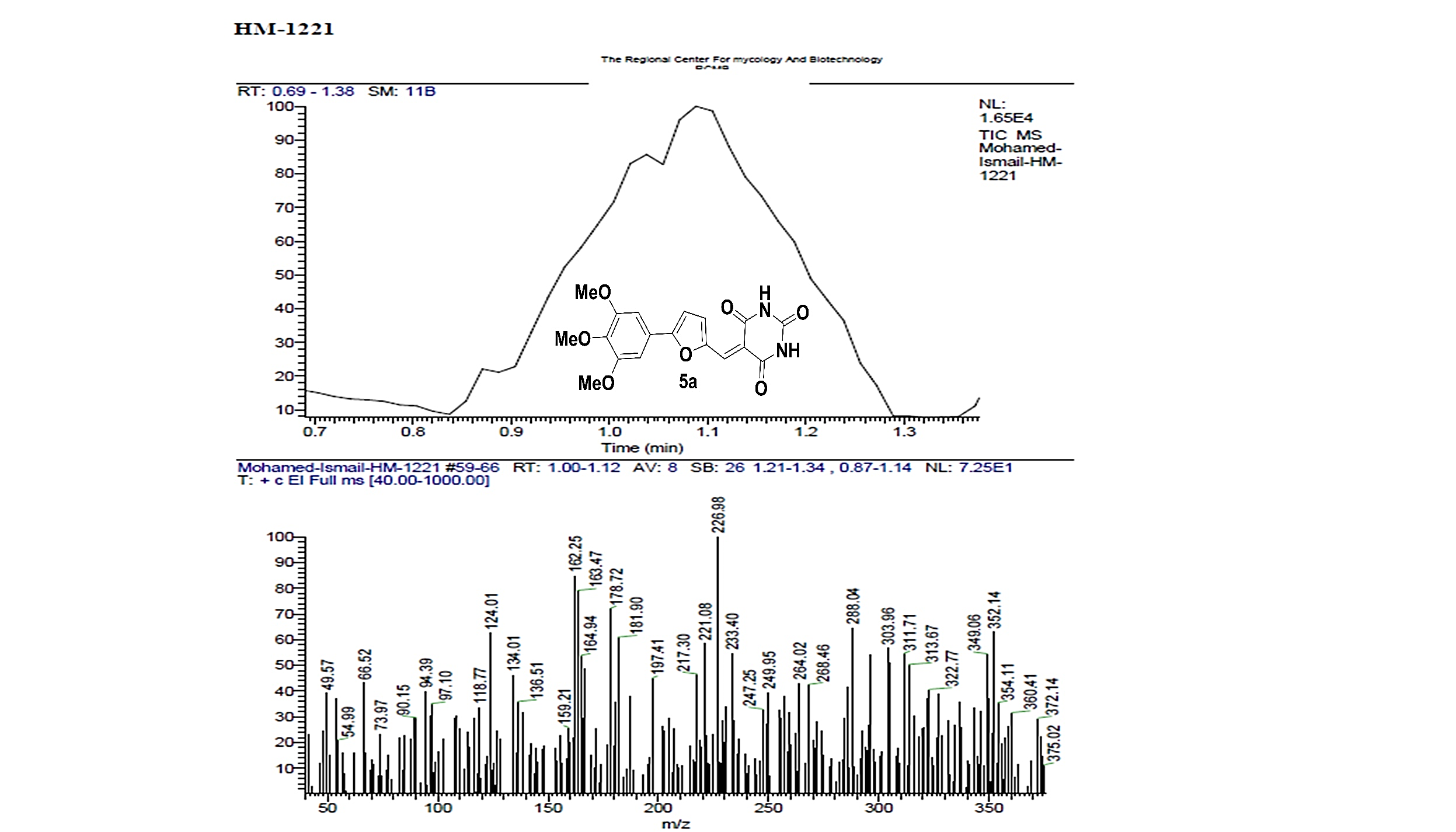
**

**Mass Spectrum of Compound HM-1221**


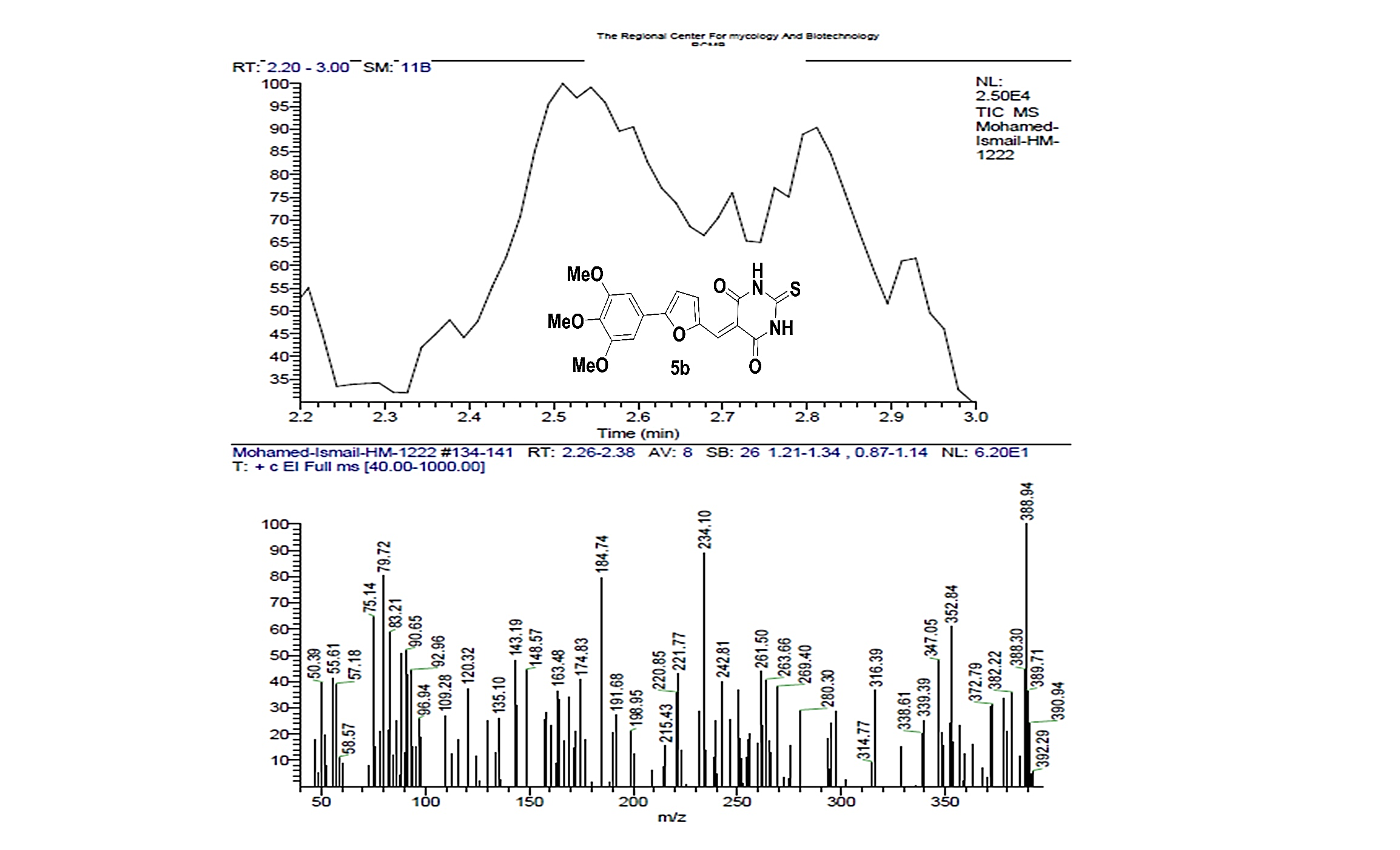


**Mass Spectrum of Compound HM-1222**


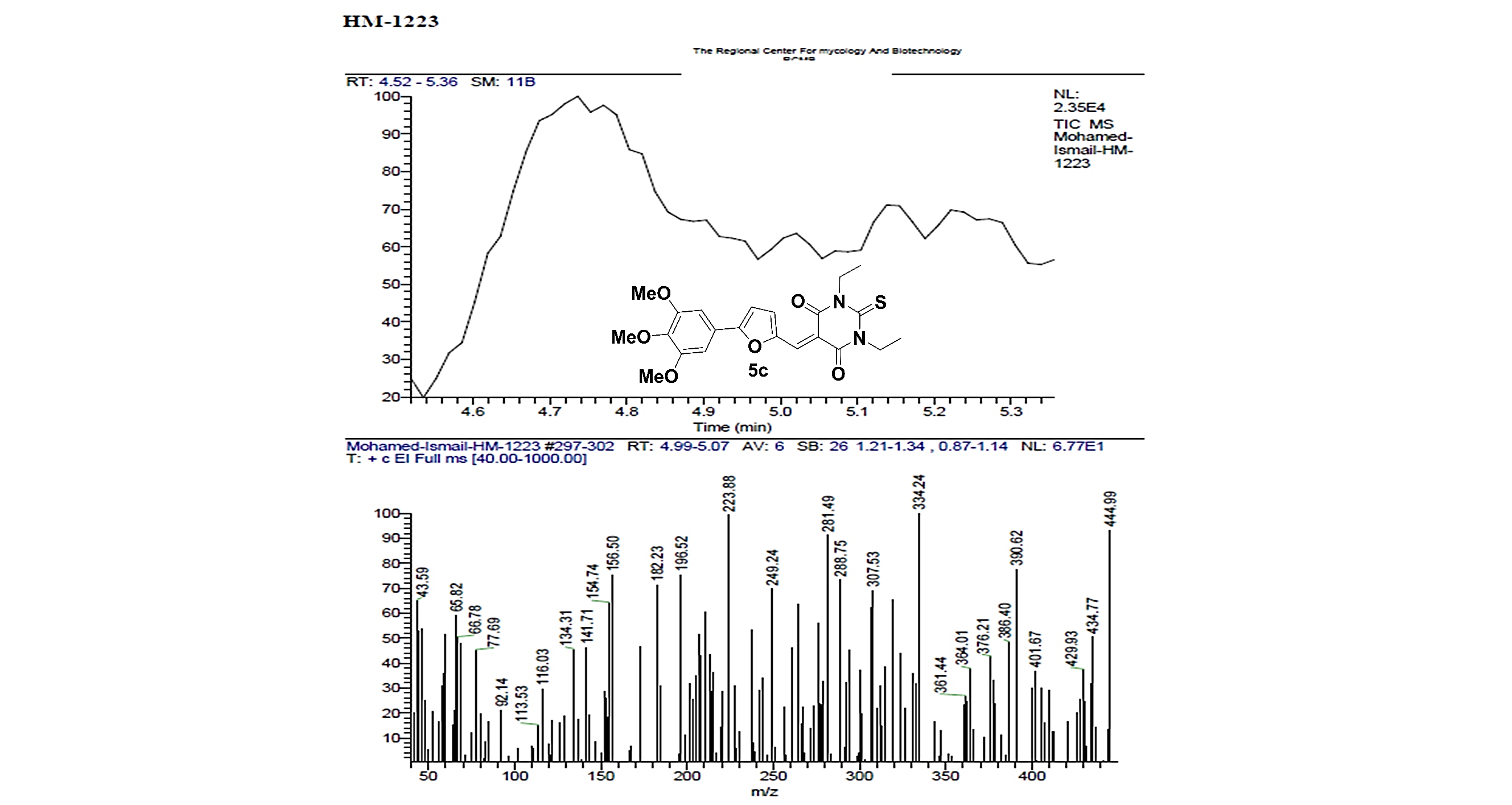


**Mass Spectrum of Compound HM-1223**


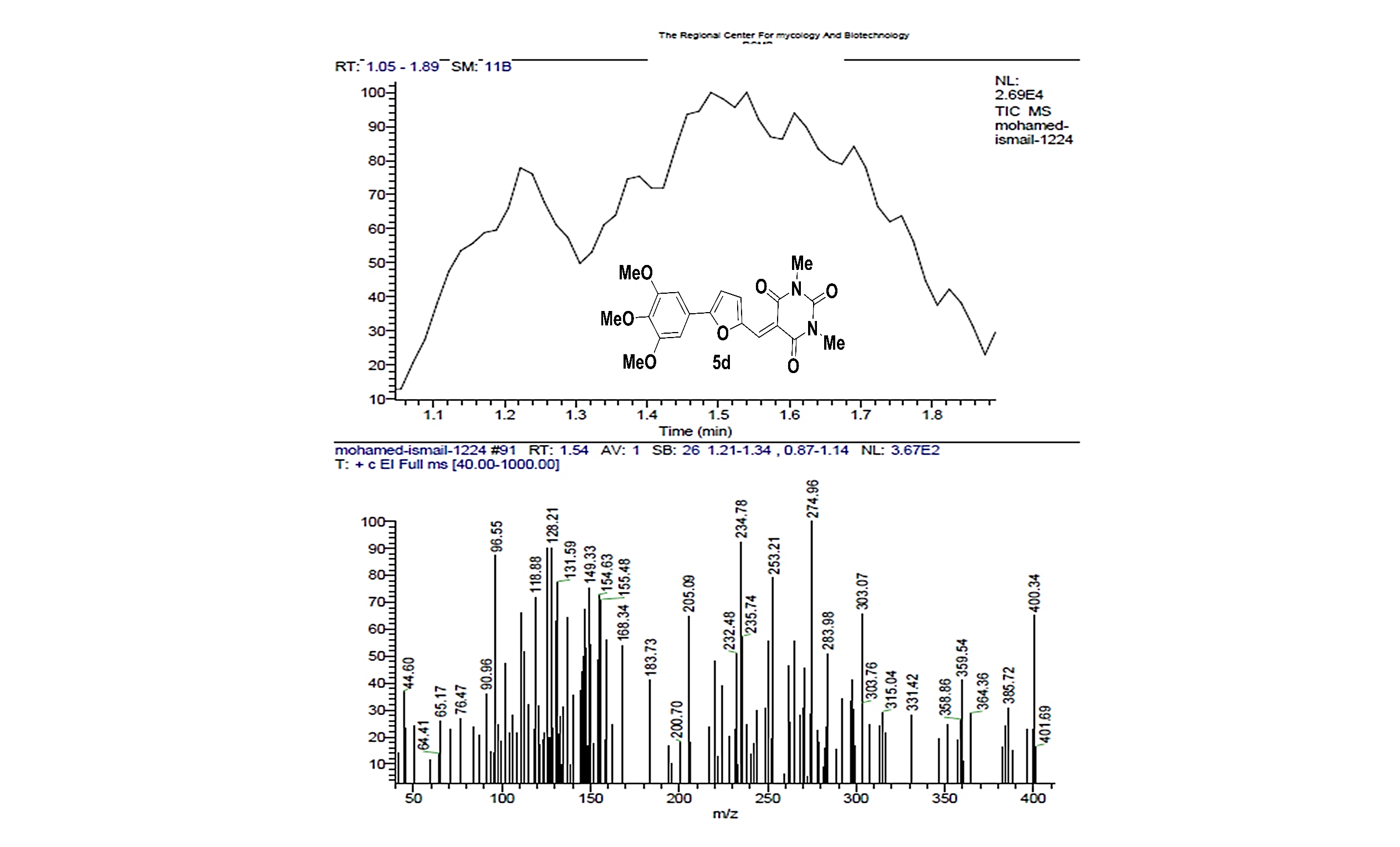


**Mass Spectrum of Compound HM-1224**
